# Measuring Realistic Emotional Perception With EEG: A Comparison of Multimodal Videos and Naturalistic Scenes

**DOI:** 10.1111/psyp.14765

**Published:** 2025-01-27

**Authors:** Andrew H. Farkas, Matthew C. Gehr, Han Jia, Dean Sabatinelli

**Affiliations:** ^1^ Department of Psychology University of Florida Gainesville Florida USA; ^2^ Department of Psychology University of Georgia Athens Georgia USA; ^3^ Department of Neuroscience University of Georgia Athens Georgia USA

**Keywords:** Bayesian, emotion, LPP, scenes, ssVEP, videos

## Abstract

Emotional experiences involve dynamic multisensory perception, yet most EEG research uses unimodal stimuli such as naturalistic scene photographs. Recent research suggests that realistic emotional videos reliably reduce the amplitude of a steady‐state visual evoked potential (ssVEP) elicited by a flickering border. Here, we examine the extent to which this video‐ssVEP measure compares with the well‐established Late Positive Potential (LPP) that is reliably larger for emotional relative to neutral scenes. To address this question, 45 participants viewed 90 matched pairs of realistic videos and scenes. Consistent with prior work, reduced 7–8 Hz ssVEP amplitude was evident during emotional relative to neutral videos. However, this reduction in power was not specific to the driving frequency of 7.5 Hz, and in fact, Fourier transformation analyses limited to 7.5 Hz were not modulated by video content. Still, at the group level, the video‐driven reductions in 7–8 Hz power and LPP modulation by scenes produced similarly large valence effects, and both measures strongly correlated with arousal ratings. Consistent with previous research, the scene‐LPP was sensitive to specific emotional contents (erotica and gore) somewhat inconsistent arousal ratings. In contrast, the video‐driven oscillation modulation did not show this content sensitivity and was better explained by individual arousal ratings per video clip. In sum, these results show that the 7.5 Hz flickering‐border paradigm does not index emotional engagement with video stimuli, yet emotional videos do evoke robust decreases in 3–10 Hz oscillatory power that is somewhat distinct from emotional modulation of the scene‐evoked LPP.

Matched emotional video and scenes evoke large EEG responses compared with neutral content within‐participant. Our findings align with previous research indicating that video modulation of power around the evoked 7.5 Hz ssVEP frequency (7–8 Hz) serves as a reliable emotional measure. However, further analyses reveal that this effect is attributable to a general decrease in power across the 3–10 Hz frequency range.

## Impact Statement

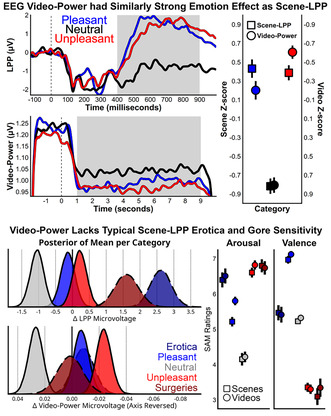

## 1 Introduction

Real‐world emotional experiences involve the perception of dynamic multisensory information, yet most affective neuroscience research evokes emotional states with unimodal stimuli such as emotional photographs of naturalistic content often called scenes. Multimodal videos would provide a more realistic perceptual experience, but the complexity of the medium has presented multiple challenges for EEG research. Historically, there has been a lack of video sets suited for electrocortical research, although realistic video sets have recently become available that are balanced for low‐level features (Ack Baraly et al. 2020; Di Crosta et al. 2020; Sabatinelli, Farkas, and Gehr 2024). However, even with ideal video sets, analyzing EEG data during video perception is challenging, as videos are not well‐suited for the primary method of isolating electrocortical activity, in which multiple trials are averaged to form event‐related potentials (ERPs; Fabiani, Gratton, and Federmeier 2007). In video perception, there is no single event, because the dynamic visual and auditory information shifts rapidly, and evokes a continuous stream of overlapping electrocortical amplitude shifts. There are analysis techniques aimed at resolving this issue, such as multivariate temporal response functions (mTRF) in which measurable stimulus changes such as visual movement or auditory intensity are mapped onto EEG data (Crosse et al. 2016). However, it is not straightforward to quantify and map the perceptual (or emotional) changes of interest that might occur over the course of even a brief video.

For these reasons, evocative naturalistic scenes are more commonly employed in affective neuroscience. Decades of research have established that emotional scenes reliably modulate many physiological responses (Bradley, Sambuco, and Lang 2022). The most replicated ERP that shows sensitivity to emotional content is the slow‐wave Late Positive Potential (LPP; Cacioppo et al. 1994) visible as a positive amplitude over central‐parietal sensors beginning roughly 400 ms after scene onset, with a long duration. The LPP is reliably larger for emotional scenes relative to neutral content. Functional MRI studies suggest that the LPP reflects an aggregate of widespread activity across many regions involved in vision, attention, and emotion manifested as a large positive voltage central parietal channels (Liu et al. 2012; Sabatinelli et al. 2013). Within‐participant effects of the LPP are consistently large; a significant effect of emotion can be found in nearly every participant given enough trials (Schupp, Flösch, and Kirmse 2023; Schupp and Kirmse 2022, 2021). In video research, such a stable and well‐defined EEG measure is not available. Thus, emotional video EEG research has used complex decoding methods (for a review, see Jafari et al. 2023), or analyses of naturally occurring oscillations such as changes in amplitude of alpha and beta oscillations (Eisenbarth et al. 2024; Romeo et al. 2022; Shestyuk et al. 2019; Simons et al. 2003).

However, recent work suggested that the emotional intensity of a video was strongly related to a continuous electrocortical signal evoked by a flickering border surrounding each video (Sabatinelli, Farkas, and Gehr 2024). The flickering border causes visual cortical regions to oscillate at the driven frequency, which can be recorded as a steady‐state visually evoked potential (ssVEP; Norcia et al. 2015). This method is useful for social and affective neuroscience because the amplitude of ssVEP amplitude can index selective attentional engagement of visually complex stimuli (Wieser, Miskovic, and Keil 2016). So whereas the LPP is thought to be generated from a collection of brain regions, the ssVEP is thought to reflect visual area activity more specifically that can be modulated by emotion and attention. For example, flickering emotional scenes evoke larger ssVEP amplitudes than neutral scenes, presumably because more neural resources are devoted to processing the emotional content (Bradley, Keil, and Lang 2012). If the driving frequency is instead evoked with a competing stimulus (such as an overlay of flickering‐dots on a static scene), then a decrease in ssVEP amplitude is evident for emotional content because they reduce engagement with the flickering dots (Deweese et al. 2014; Deweese, Müller, and Keil 2016; Müller, Andersen, and Keil 2008). A benefit of the ssVEP paradigm is that the signal‐to‐noise ratio is often large relative to ERPs because of the stability of the neural oscillation, thus allowing for strong filtering of the data around the driving frequency. This is ideal for video stimuli because the measured ssVEP should more clearly reflect video engagement rather than lower level perceptual properties of the video. By presenting a flickering border surrounding each of 45 video clips, we found that a narrow‐band Hilbert transformation of the ssVEP frequency decreased in amplitude to emotional videos for nearly all participants (Sabatinelli, Farkas, and Gehr 2024). Importantly, the amplitude was strongly correlated with self‐reports of emotional arousal, and not correlated with video motion, brightness, loudness, or visual complexity. The emotional modulation of the oscillation amplitude was also larger and longer in duration than has been reported for emotional scenes (Deweese et al. 2014; Deweese, Müller, and Keil 2016).

While emotional modulation of the ssVEP to videos was robust in our initial study, it is unclear to what extent the dynamic nature of the medium affects electrocortical reactivity relative to the notably strong emotional modulation of the LPP to emotional scenes. Pragmatically, it is possible that a video‐ssVEP paradigm might provide a larger and more reliable index of emotional modulation than a scene‐LPP paradigm. Alternatively, videos may recruit and maintain more generalized brain reactivity to such an extent that emotional modulation may be comparatively weak and variable relative to what is engaged in a static scene paradigm.

Here, we address this research question by presenting content‐matched videos and scenes within a group of participants to directly compare the video‐modulated ssVEP to the scene‐evoked LPP. From an expanded set of 90 curated video clips, a matched scene was selected from the most representative frame from each video, or a closely similar scene if no individual frame was appropriate. In the first set of analyses, affective ratings and Z‐scored electrocortical measures were compared using traditional statistics to determine whether these measures differed meaningfully between the paradigms. This was supplemented with more resolved analyses using Bayesian multilevel models, which allowed for a thorough description of the results at the raw microvolt scale. The models were also used for well‐powered post hoc tests of individual stimulus contents that were regularized and corrected for multiple comparisons. Based on relevant prior research, we hypothesized that emotional videos would be rated as more arousing than their matched scene counterparts (Detenber, Simons, and Bennett 1998; Simons et al. 1999), creating a larger distribution of values. Members of the research team differed in their opinions of which EEG measure would produce a larger emotional effect based on which measure would have a more consistent signal to noise ratio, and thus, we considered the outcome to be equally likely in either direction.

## 2 Methods

### 2.1 Participants

Fifty participants were recruited from the University of Georgia undergraduate student body and compensated with psychology course credit. All participants had to be 18 years or older, with normal to corrected‐to‐normal vision. Participants' data were excluded if the EEG preprocessing stream removed over 50% of trials from the pleasant, neutral, or unpleasant categories from either the scene or video series. Of the 50 participants, 45 were included in the final analyses. One participant discontinued the study because of an unrelated illness. Another participant's data were lost because of a computer crash. Finally, three participants were excluded because of an excess of artifact contaminated trials, described below. Of the remaining 45 participants, the mean age was 19.05 (SD = 1.03). Self‐identified sex was 32 females and 13 males. Self‐identified race/ethnicity was 2 Black, 7 Asian, 1 Hispanic, 2 Indian, 1 Middle‐Eastern, 2 Multiracial/Multiethnic, and 29 White. One participant chose not to identify their age or racial/ethnic identity. All participants were given informed consent before the study that had been approved by the University of Georgia Human Subject Institutional Review Board.

### 2.2 Stimuli

The video stimuli were curated with the same objectives described in our previous video study (Sabatinelli, Farkas, and Gehr 2024). Unlike the previous study that used 45 video clips shown twice, this study featured 90 videos. Mutilation content of graphic injuries is a common stimulus category for emotional scene research. However, mutilation videos are difficult to find and gather ethically. Thus, videos of surgeries are used, which have similar gory depictions as mutilation content. Surgery videos were selected from official sources such as hospital online accounts that had posted the videos for training purposes. Overall, video content was selected in an attempt to depict a diverse range of people and cultures. Each was selected for clarity such that the situations depicted are understandable from the start of the video. Realism was also a point of emphasis such that there was genuine elements and audio. This includes the absence of professional film elements such as music, composition, or lighting. Some neutral videos were filmed by the research team in public places such as markets and city streets. Each video was 10 s in duration with a single‐lens perspective at roughly eye‐level. From the videos, 90 frames were selected to serve as scene stimuli, in order to match the content as closely as possible.

A matching scene was selected for each of the 90 videos. This was accomplished by picking the most representative frame from each video by a member of the research team based on experience from past scene studies. The choices were confirmed by the other the authors. For five videos, there was not a frame that effectively depicted the content (e.g., a video of a baby otter harmlessly falling into the water and being cuddled by a parent). For these five videos, an outside source was used to provide an equivalent scene that matched the video content (e.g., a baby otter and parent cuddling). The scenes' resolution matched the videos at 960 by 540 pixels. Visual complexity was approximated with JPEG file size when maintained at 90% quality (Donderi 2006). These low‐level scene features did not differ between pleasant, neutral, and unpleasant content using the same ANOVA threshold (*p* > 0.2).

Multiple procedures were performed to control as many of the video low‐level visual and auditory features as possible. Videos were selected and edited to have roughly similar levels of brightness and loudness. Loudness was measured with a BAFX 3370 Digital Sound Level Meter placed at ear level at the participant's chair. Average brightness of the videos was measured by converting the video frames to grayscale images and measuring the average pixel value from white (255) to black (0). Overly bright or dark videos were adjusted such that they were as close to the default gray color used by psychopy and common in visual science experiments (Peirce et al. 2019; RGB value 148,148,148). The resolution of each video was 960 by 540 pixels and were presented at 24 frames per second. Lastly, visual movement was assessed by the changes in individual pixel brightness from frame to frame. To achieve this, each frame was converted to a grayscale image with each pixel ranging from white (255) to black (0). The differences for each pixel was then found from the current frame to brightness values of the subsequent frame. The average change in brightness for each individual pixel was the quantified unit of movement. This was repeated for all frames to get a broad measure of movement for each video. The procedure used the Magick R package version 2.7.3 (Ooms 2021). Standard ANOVAs between emotional categories confirmed that all the low‐level video features were equivalent across the pleasant, neutral, and unpleasant videos (*p* > 0.2).

A limitation of our previous study was that there was no baseline period as the video onset occurred simultaneously with the flickering border that evoked the ssVEP (Sabatinelli, Farkas, and Gehr 2024). Scene ssVEP studies have overcome this by beginning each presentation with a scrambled version of the scene, in which the pixels are randomly rearranged, depicted with the competing flickering stimulus before switching to the actual scene (Deweese et al. 2014; Deweese, Müller, and Keil 2016). This creates a baseline for the content‐modulated ssVEP to diverge from that potentially improves interpretability and statistical power. This approach was emulated here by adding a brief segment of randomly moving pixels and audio to the beginning of each video clip. Random frames were taken from each video, and the pixels were rearranged within each frame. These frames were then added to the start of each video until there was 2 to 4.5 s of additional random movement. White noise was added to each scrambled video that matched the average decibel level of the actual video. The scrambled video and audio was smoothly transitioned to the actual video over 0.5 s (or 12 frames) to prevent an abrupt onset. This was done by weighting the video pixels and audio tracks in a gradient from random video to the actual video. All video editing was accomplished with the “moviepy” Python package.

### 2.3 Procedure

**FIGURE 1:**
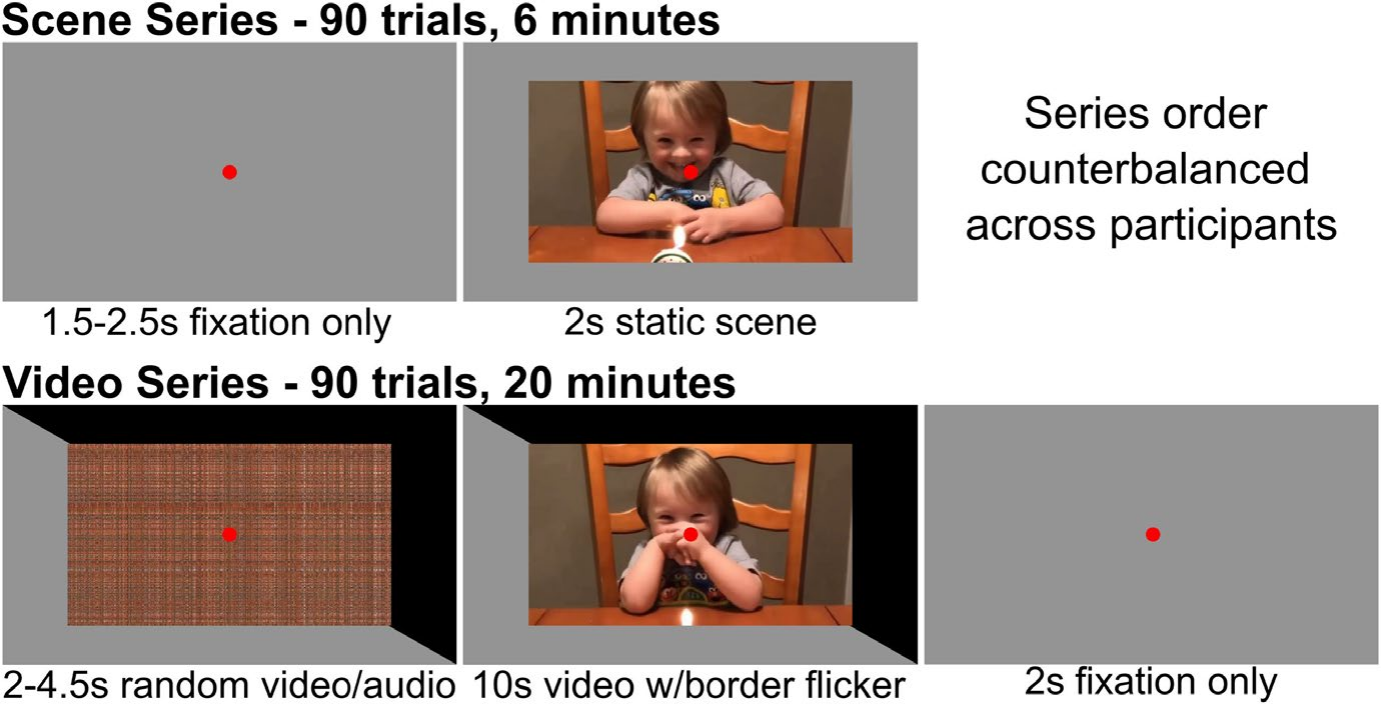
Each participant saw the scenes and video series as depicted here. Whether the scene or video series came first was counter‐balanced between participants. For the video series, the entire border flickered from gray to black. The border changed to black every 8th screen refresh, causing a 7.5 Hz flicker that served as the ssVEP driving frequency.

After signing the informed consent document, participants were led into an electrically shielded chamber for EEG recording. While seated, participants were fit with the 128‐channel EEG cap. Participants sat with the eyes approximately 120 cm from a 60 Hz LG UltraGear QHD HDR monitor (27GN800‐B), on which the scenes and videos were presented in landscape orientation over 7 by 12 in., resulting in 8.5 by 14° of visual angle. Each participant saw the scene and video series as depicted in Figure 1. Whether participants saw scenes or videos first was counterbalanced between participants. The order of stimulus presentation did not result in a statistical difference for the LPP or oscillation‐power as measured with repeated measures ANOVAs (*p* > 0.15). Two pseudorandomized orders were produced for videos and scenes such that pleasant, neutral, and unpleasant content was balanced across each series. The two orders were also counter balanced between participants such that there were four possible presentation paradigms (videos first/random order 1, scenes first/random order 1, videos first/random order 2, and scenes first/random order 2). The order of the content also did not produce any statistical differences (*p* > 0.2). After the first series of stimuli, the research team checked in with the participants briefly and then proceeded to the next series.

Participants were asked to maintain fixation on a small red dot in the middle of the screen throughout the entire series, including fixation periods. Participants were also asked to limit blinking while content was on the screen. Scenes were presented for 2 s with a random interstimulus interval between 1.5 and 2.5 s. Video trials began with a 2‐s fixation followed by 2 to 4.5 s of random video and audio generated as described earlier. The 2‐s break from flickering was to aid in participant comfort, while the 2 to 4.5 s was varied to prevent possible prediction of stimulus onset. During video presentation, the entire outer section of the video monitor changed from gray (RGB value of 148,148,148) to black (maximum value of 0, 0, 0) every 8th screen refresh creating a 7.5 Hz flickering stimulus. Piloting before our previous study (Sabatinelli, Farkas, and Gehr 2024) suggested that gray to black minimizes eye strain for participants and leads to similar ssVEP amplitudes as gray to white. Task engagement is monitored during EEG data collection by the research team by visualizing real‐time EEG signals.

After EEG data collection, the cap was removed and participants moved to a separate quiet room to rate each scene/video on a computer using an adapted version of the Self‐Assessment Manikin (SAM; Bradley and Lang 1994). Participants were first read a slightly modified version of the instructions for the original SAM protocol to explain how to rate the stimuli on their experienced valence (pleasantness) and arousal when they first viewed the content. Ratings were made with a draggable slider that was positioned underneath each stimuli and the SAM figures. The scale ranged from 1 (completely pleasant or arousing) to 9 (completely unpleasant or unarousing). Intermediate values could be made in integer increments between 1 and 9 with 5 being labeled as neutral. Each stimulus was shown for the pleasantness scale first and again for the arousal scale. The slider defaulted to the 5 position, but required participants to click on the line before clicking a button to advance to the next trial. The scales were reversed for statistical analyses and figures. For comfort and time constraints, participants were not required to watch the entirety of each video. An example trial was shown to participants with the researcher present, and then the participant was left in the room to complete their ratings. Participants were given headphones to hear each video. After the ratings, participants were given a brief postexperiment questionnaire for feedback to the research team. Participants were then debriefed and excused from the study.

### 2.4 EEG Acquisition and Processing

The EEG data were recorded with a BioSemi ActiveTwo EEG 128‐channel cap (BioSemi Amsterdam, Netherlands) using the standard procedure of placing the Cz sensor halfway between the nasion and inion according to the 10–20 layout. The system used two common model electrodes (Common Mode Sense and Driven Right Leg), after which the data were re‐referenced to the average‐reference in later processing. Data were recorded at a 512 Hz sampling rate with no filters (https://www.biosemi.com/faq/adjust_filter.htm). Site preparation with electrolyte gel continued until sensor offsets were between 50 and −50 mV.

The EEG data were processed using EMEGS (emegs.org; Peyk, DeCesarei, and Junghöfer 2011), an open‐source MATLAB software package. Graphical interface windows and all code used can be seen on this project's Open Science Framework Webpage (https://osf.io/gx243/), which is linked to a Github code repository (https://github.com/AndrewHFarkas/Pic_Vid). The EMEGS software was used to implement the SCADs procedure, which is a semiautomated pipeline for detecting and correcting artifacts in dense‐array EEG and MEG data (Junghöfer et al. 2000). Scene‐evoked EEG data were extracted from 125 ms prior to 2000 ms after scene onset, whereas video EEG data were extracted from 2 prior to 10 s after video onset/video baseline offset. A smaller epoch could have been selected for both measures to retain slightly more trials, but it did not change the results or interpretation. The larger epochs allow for visualizing the baseline period for the ssVEP (Figure 4), and it is sometimes useful for researchers to study later portions of the LPP beyond 1 s from onset. Both measures were low‐pass‐filtered with an 18th‐order Butterworth filter using a passband of 30 Hz (3 dB point) and stopband of 40 Hz (45 dB attenuation). Scene LPP data were high‐pass filtered using a 4th‐order Butterworth with a passband of 0.1 Hz (1 dB point) and stopband of 0.05 Hz (18 dB attenuation). Because we were mainly interested in the 7.5 Hz ssVEP frequency, the video EEG data used a more aggressive high‐pass Butterworth 3rd‐order filter with a passband of 3 Hz (1 dB point) and stopband of 1 Hz (18 dB attenuation).

To identify trials and sensors that were contaminated by artifacts, the SCADS method forms distributions for trials by sensors from a composite measure of maximum amplitude, first‐derivative, and standard deviation indices. The final sample had on average 76.3 retained scene‐LPP trials and 72.9 video trials (grand totals being 3441 scene versus 3358 video trials). Noisy sensors are removed and replaced with by a spherical spline that weights the closest and least noisy sensors. To isolate the ssVEP signal of interest, a custom function was used on the video EEG data. This function filters the data 0.5 Hz above and below the target frequency of 7.5 Hz, and then applies a Hilbert transformation to derive the oscillatory amplitude over the duration of each video epoch. Of note, during peer review, it was discovered that the quantification of the ssVEP with a narrow‐band Hilbert transformation is altered by EEG‐power within the filter between 7 and 8 Hz. Because the results therefore reflect a change in ssVEP amplitude outside the driven frequency of 7.5 Hz, we refer to this Hilbert‐derived video measure as oscillation‐power from this point forward. Additional Fourier analyses have been included to provide evidence of this underlying effect, and the implications are expanded upon in the discussion.

Nine sensors were used to estimate the scene‐LPP and video oscillation‐power. For the LPP, the nine sensors occupied the typical LPP central‐parietal region centered around the CPz sensor used in our previous studies (CCP1h, CP1, Cz, CPP1h, CPz, CPP2h, CP2, CCP2h, and Pz; Farkas, Oliver, and Sabatinelli 2020; Farkas, Wanger, and Sabatinelli 2021; Farkas and Sabatinelli 2023). For oscillation‐power, the nine chosen sensors centered around Oz (over primary visual regions) where ssVEP amplitude is largest and in line with our previous video study (POz, O9/I1, O1, OI1h, Oz, Iz, O2, OI2h, O10/I2; Sabatinelli, Farkas, and Gehr 2024). The LPP time window was 400 to 900 ms postscene onset, consistent with previous studies (Farkas, Oliver, and Sabatinelli 2020; Farkas, Wanger, and Sabatinelli 2021; Farkas and Sabatinelli 2023). The video window was from 1 to 9 s after video onset, to avoid oscillatory influences of onset and offset, as in our previous video study (Sabatinelli, Farkas, and Gehr 2024). The LPP was baselined with data across the 100 ms prescene onset. Baselining the oscillation‐power (e.g., the 500 ms prevideo onset) resulted in equivalent valence category differences in magnitude, but lower statistical power due to elevated EEG noise in the transitional video baseline period. For this reason, we do not present the oscillation‐power results as deviated from prestimulus baseline.

During peer review, it was discovered that naturally occurring oscillation changes surrounding the 7.5 Hz driving flicker are most likely driving the video effects. We confirmed this by performing a Fourier Transformation to plot the average power using the same sensors and epoch for each participants and video category. We plot the power spectrum in Figure 8. The average power was found for the ssVEP 7.5 Hz frequency, from 7 to 8 Hz, which was included in the initial Hilbert‐derived power, and a exploratory window from 3 to 10 Hz based on a visualization of Figure 8. Each power measure was Z‐scored within‐participant as described in the group‐level statistics section below. The 7.5 Hz ssVEP effect size did not change when the ssVEP was further isolated using a sliding window analysis, which was implemented with the MATLAB Freq Tag toolbox (Figueira et al. 2022).

### 2.5 Group‐Level Statistics

Initial statistics were performed on the average of each emotional category by participant, with additional analyses by video/scene stimulus, in which all participants were averaged. The 90 trials/stimuli each belonged to one of three predetermined categories of pleasant, neutral, unpleasant content. Within‐each participant, the average cortical amplitude and SAM ratings was calculated per category for videos and scenes. The three category amplitudes for LPP and ssVEP were then Z‐scored within participant to enable comparison. The distribution of category raw‐microvoltage versus Z‐scored amplitudes can be seen in Figure S1. A factorial ANOVA was then performed in which category and stimuli type (scenes or videos) were within‐participant factors. This ANOVA was used for emotion ratings as well as the EEG amplitudes. This was followed up with repeated measure ANOVAs for each stimuli type independently, and then results were broken down further with paired *t*‐tests. For the by‐stimulus analyses, the amplitudes were again Z‐scored within‐participant but without averaging the trials into categories. These Z‐scores per stimulus were then averaged across participants, resulting in one average value for each of the 90 videos and scenes. The stimuli were then correlated with average arousal rating (which has a strong association with scene‐LPP and video oscillation‐power; Farkas and Sabatinelli 2023; Sabatinelli, Farkas, and Gehr 2024). To assess whether the strength of the linear relationships differed between stimulus types, a linear regression analysis was done on the same data with stimuli type modeled as an interacting effect with the arousal rating predictor.

### 2.6 By‐Trial Multilevel Bayesian Analyses

Bayesian Multilevel models were used to analyze the measures on their original scales and achieve more robust statistical descriptions and inferences. It is commonly recommended that results are presented in their original units for open‐science and replication efforts for neuroimaging (Keil et al. 2014; Niso et al. 2022; Pernet et al. 2020; Picton et al. 2000) and psychophysiological recording (Blumenthal et al. 2005; Boucsein et al. 2012; Steinhauer et al. 2022). The resulting Bayesian probability distributions (posteriors) are descriptive as they show microvolt values that are the most likely as opposed to interval/range of possible values. Beyond this, a primary benefit of a multilevel Bayesian model is that it efficiently balances explanation and prediction against overfitting and false‐positives (Gelman 2013; Gelman, Hill, and Yajima 2012). This allows for more credible granular results from more complex models. The models have a by‐default form of regularization, which makes it more predictive for future uncollected datasets, as if it were tuned by a cross‐validation algorithm (Gelman 2013; McElreath 2020). In this kind of model, parameters (such as the mean for each participant) are estimated simultaneously with a higher level distribution (the estimated distribution of all participant means). The higher level distribution becomes a regularizing prior that corrects the most extreme posteriors by pulling them toward the center in a process known as shrinkage. Thus, individual parameters are both informed and constrained by the group. The last noteworthy benefit is that the results are a by‐trial generative model that can be used by future research teams to simulate outcomes or for power analyses.

Two models are presented that were fit with the software package Stan (Stan Development Team 2024). Here, it was used to estimate posteriors via a modified form of Hamiltonian Monte Carlo sampling (Duane et al. 1987; Stan Development Team 2024). This algorithm is similar to other Markov Chain Monte Carlo methods, but appears to be more accurate and computationally efficient for complex models when many parameters are estimated (McElreath 2020; Stan Development Team 2024). A total of 80,000 posterior samples were taken from each posterior using eight sampling chains of 10,000 samples, which was above what was needed for accurate posteriors but resulted in clearer figures. Trace plots and the metrics of “*R*‐hat” (< 1.01) both indicated that the chains converged and the posteriors were accurately estimated. The summary statistics for all posteriors can be found in the supplemental materials.

Models were built according to best practices (Gelman 2013; McElreath 2020). This involved building from simpler to more complex models with simulated data. Thus, the two models presented here are not the only models fit to the data. However, the other models did not add to the interpretability of the results. Additionally, the two chosen models have the highest cross‐validation accuracy as measured with the Pareto Smoothing Importance Sampling Leave One Out method (PSIS‐LOO; Vehtari, Gelman, and Gabry 2017). This suggests that the models balance complexity with prediction accuracy best and would be the most accurate in forecasting future data. The *k* diagnostic statistics indicate that the cross‐validation results were not biased by outliers, so the cross‐validation measure expected log point density (ELPD) would lead to equivalent interpretations as traditional LOO or k‐fold cross‐validation algorithms. The other models and their summary statistics can be found on this project's Open Science Framework page (https://osf.io/gx243/). All models were fit on single‐trial data which is also recommend for these type of analyses (McElreath 2020). Posterior results are reported with their median value as well as the inner 95% of samples known as credibility intervals (CI).

The first model predicted single‐trial amplitudes using participants and stimuli as predictors. The model is specified in the following equations which also act as a transparent listing of statistical assumptions.

**Model 1: multilevel participant and stimuli predictors**

Where (i) indexes all observations, (j) indexes all 45 participants, and (k) indexes all 90 stimuli.

Amplitude is a vector of observed cortical amplitudes (either LPP or ssVEP). Par is a vector of a participant indices (1 to 45) for each Amplitude. Stim is a vector of stimuli indices (1 to 90) for each Amplitude.

**Linear Model (Likelihood)**

(1) $$\text{Amplitud}e_{\left[i\right]}∼\text{Normal}\left(\mu_{\left[i\right]},\sigma_{\left[\mathrm{Par}\left[i\right]\right]}\right)$$

(2) $$\mu_{\left[i\right]}=\beta_{1\left[\mathrm{Par}\left[i\right]\right]}+\beta_{2\left[\text{Stim}\left[i\right]\right]}$$

**Adaptive Regularizing Priors**

(3) $$\beta_{1\left[j\right]}∼\text{Normal}\left(\bar{\mathrm{Par}},\text{σPar}\right)$$

(4) $$\beta_{2\left[k\right]}∼\text{Normal}\left(0,\text{σStim}\right)$$

(5) $$\sigma_{\left[j\right]}∼\text{Normal}\left(\bar{\sigma },\tau \right)$$

**Uninformative Weak Priors**

(6) $$\bar{Par}∼\text{Normal}\left(\bar{\text{Amplitude}},2\cdot \mathrm{SD}\left(\text{Amplitude}\right)\right)$$

(7) $$\sigma \text{Par},\sigma \text{Stim},\bar{\sigma },\tau ∼\text{HalfNormal}\left(0,2\cdot \mathrm{SD}\left(\text{Amplitude}\right)\right)$$

Line 1 states that each amplitude comes from a normal distribution with a mean (*mu*) and standard deviation (*sigma*). *Sigma* can be thought of as the estimated per‐trial residual error. The indices show that each participant gets their own sigma. This led to better cross‐validation accuracy and makes the model more comparable to Model 2. It is also preferable because it is common for a physiological measure's error to be different between participants. Line 2 depicts a deterministic linear model in which *mu* is the sum of the current participant's mean (*Beta1*) and the current stimuli (*Beta2*). Line 3 is what makes this a multilevel model; it specifies that each participant comes from a higher‐level normal distribution of participants. The higher level distribution takes two parameters: the estimated mean for all participants (*ParBar*) and the standard deviation of participants (*sigmaPar*). Line 4 invokes the same logic; the 90 stimuli are drawn from a higher‐level distribution of stimuli. However, the mean of this distribution is set at zero so that the effect of stimuli deviates from the current participant's mean. The last adaptive prior on Line 5 constrains each participant's amplitude standard deviation with a normal distribution that takes estimated mean standard deviation (*sigma bar*) and a separate standard deviation parameter (*tau*). Lastly, Lines 6 and 7 specify weak priors for the outstanding parameters of interest. To enable the models to be compared for the LPP and ssVEP, the weak priors had to be relatively similar in scale between the measures. To do this, the empirical mean (*AmplitudeBar*) and standard deviation (SD(Amplitude)) of the data were used. By multiplying the standard deviation by 2, this created a flat/wide prior distribution relative to the data that was actually collected. A weak prior makes sure the relevant posteriors are based mostly on the data that was collected. Multiplying by 3 or greater has no meaningful effect on the results. Half‐normal distributions are used for the standard deviation parameters because standard deviation cannot be negative.

Model 1 was used to describe the certainty of mean amplitudes for the predetermined categories of pleasant, neutral, and unpleasant content (Figures 6 and 7). In this statistical framework, this is done by averaging the posterior samples for each stimuli (*Beta2*) that belonged to each of the three valence categories. To find the statistical difference between two posteriors, the samples can be contrasted by subtracting each sample from the other. This was used to find the posterior for the difference in brain reactivity between emotional and neutral content. To visualize between‐participant effects and how they differed between the LPP and ssVEP, the posterior for each participant's mean (*Beta1*) was also plotted. For an estimate of the distribution of participants, 80,000 samples were taken from a normal distribution for each posterior sample of the estimated mean of participants (*ParBar*) and the estimated standard deviation of participants (*sigmaPar*). To compare the signal‐to‐noise ratio (SNR) of emotional vs. neutral stimuli between the ssVEP and LPP, the posterior for the mean of emotional trials was contrasted with posterior mean of neutral trials divided by the posterior for average single‐trial error (*sigmaBar*).

(8) $$\text{Model}\;1\;\mathrm{SNR}=\frac{\bar{\beta_{2}\left[\text{emotional stimuli}\right]}-\bar{\beta_{2}\left[\text{neutral stimuli}\right]}}{\bar{\sigma }}$$

Note that the signal defined in Line 8 is the difference between two trials, while the noise is the residual error for one trial. The signal posterior is the same as the white curve depicted Figure 6. The SNR posteriors between the video oscillation‐power and LPP were contrasted to understand the probability that one measure was larger.

It has been demonstrated that the LPP is particularly sensitive to erotic and mutilation content (Farkas, Oliver, and Sabatinelli 2020; Farkas and Sabatinelli 2023; Weinberg and Hajcak 2010). To visualize if there was statistical evidence for that sensitivity for the scene‐LPP and video oscillation‐power in the current data, the stimuli posteriors (*Beta1*) were rearranged such that posterior samples of the 8 erotic and 5 surgery stimuli were separated and averaged apart from the overall pleasant and unpleasant posteriors. Concerns about post hoc statistical inference are lessened because of the multilevel regularization described above. An unequal amount of erotic and surgery stimuli are not a strong concern in this framework. Less observations will be reflected in flatter posteriors indicating there is more uncertainty given the limited data.

Whereas Model 1 predicts cortical amplitudes based on the stimuli participants viewed, Model 2 predicts cortical amplitudes based on arousal ratings without stimuli as a predictor. Comparing these models therefore tests whether stimulus content or experienced emotion arousal is more predictive of cortical reactivity.

**Model 2: multilevel amplitude and arousal bivariate distribution**

Where (i) indexes all observations and (j) indexes all 45 participants.

Amplitude is a vector of observed cortical amplitudes (either LPP or ssVEP). Arousal is a vector of observed arousal ratings aligned with Amplitude. Par is a vector of a participant indices (1–45) for each Amplitude.

**Bivariate Distribution (Likelihood)**

(9) $$\left[\begin{array}{c} {\text{Amplitude}}_{\left[i\right]}\\ {\text{Arousal}}_{\left[i\right]} \end{array}\right]∼\mathrm{MV}\;\text{Normal}\left(\left[\begin{array}{c} \mu {\mathrm{Amp}}_{\left[\mathrm{Par}\left[i\right]\right]}\\ \mu {\mathrm{Aro}}_{\left[\mathrm{Par}\left[i\right]\right]} \end{array}\right],\sum_{\left[\mathrm{Par}\left[i\right]\right]}\right)$$

(10) $$\sum_{\left[\mathrm{Par}\left[i\right]\right]}=\left[\begin{array}{cc} \sigma {\mathrm{Amp}}_{\left[\mathrm{Par}\left[i\right]\right]}^{2} & \sigma {\text{AmpAro}}_{\left[\mathrm{Par}\left[i\right]\right]}\\ \sigma {\text{AmpAro}}_{\left[\mathrm{Par}\left[i\right]\right]} & \sigma {\mathrm{Aro}}_{\left[\mathrm{Par}\left[i\right]\right]}^{2} \end{array}\right]$$

**Adaptive Regularizing Priors**

(11) $$\mathrm{\mu Am}p_{\left[j\right]}∼\text{Normal}\left(\bar{\text{μAmp}},\text{σParAmp}\right)$$

(12) $$\mathrm{\mu Ar}o_{\left[j\right]}∼\text{Normal}\left(\bar{\text{μAro}},\text{σParAro}\right)$$

(13) $$\mathrm{\sigma Am}p_{\left[j\right]}∼\text{Normal}\left(\bar{\text{σAmp}},\text{τParAmp}\right)$$

(14) $$\mathrm{\sigma Ar}o_{\left[j\right]}∼\text{Normal}\left(\bar{\text{σAro}},\text{τParAro}\right)$$

(15) $$\text{σAmpAr}o_{\left[j\right]}∼\text{Normal}\left(\bar{\text{σAmpAro}},\text{τAmpAro}\right)$$

**Uninformative Weak Priors**

(16) $$\bar{\mu \text{Amp}}∼\text{Normal}\left(\bar{\text{Amplitude}},2\cdot \mathrm{SD}\left(\text{Amplitude}\right)\right)$$

(17) $$\begin{array}{c} \text{σParAmp},\bar{\text{σAmp}},\text{τParAmp},\text{τAmpAro}\\ ∼\text{HalfNormal}\left(0,2\;\mathrm{SD}\left(\text{Amplitude}\right)\right) \end{array}$$

(18) $$\bar{\text{μAro}}∼\left(\text{Beta}\left(1.1,1.1\right)\cdot 8\right)+1$$

(19) $$\text{σParAro},\bar{\text{σAro}},\text{τParAro}∼\text{HalfNormal}\left(0,4\right)$$

(20) $$\bar{\sigma \text{AmpAro}}∼\text{Normal}\left(0,2\cdot \mathrm{SD}\left(\text{Amplitude}\right)\right)$$

Line 9 of Model 2, each observed amplitude and arousal rating is modeled as coming from a two‐dimensional normal distribution (bivariate distribution). A multivariate normal distribution takes a vector of mean parameters (here being *muAmp* & *muAro*) and a covariance matrix (uppercase *Sigma*). The indices and Line 10 show that each participant gets an estimated mean for their amplitude and arousal ratings (*muAmp* and *muAro*), and also their own covariance matrix. A more traditional multilevel regression model, in which each participant gets their own intercept and slope coefficients, was also fit to the data and can be found in the OSF page of this project (https://osf.io/gx243/). However, that model fit was worse and had worse cross‐validation accuracy because it did not account for how participants used the arousal scale differently. Another advantage of this model is that a correlation posterior can be found for each participant from each covariance matrix.

In a traditional statistical framework, this number of parameters would be likely to cause overfitting and lead to unreliable estimates. Here, because each of these parameters are estimated jointly with higher level distributions, the posteriors are regularized leading to better cross‐validation accuracy. Lines 11 through 15 specify these higher level distributions that each parameter is drawn from. Normal distributions for the standard deviation parameters were set with a lower bound of zero to prevent negative values.

To complete Model 2, weak uninformative priors were set for the outstanding parameters. As in Model 1, parameters relating to cortical amplitudes used the same empirically measured standard deviation value of the scene‐LPP and video oscillation‐power multiplied by two. The priors for arousal ratings reflect that the scale ranged from 1 to 9. A Beta distribution with two parameters of 1.1 form a nearly uniform distribution between 0 and 1. This was multiplied by 8 and increased by 1 to scale it to extend across the arousal ratings range of 1 to 9. For standard deviation values relating to arousal ratings, a half normal distribution of 4 is wide compared to how participants used the rating scale, making it a weak prior. Because the purpose of the model is to predict cortical amplitudes and not arousal ratings, the measure of accuracy was the log‐likelihood of each amplitude given arousal ratings. The log‐likelihoods were found via the normal_lpdf function implemented in the “generate quantities” section of the Stan model using the following equations for the parameters for each observation:

(21) $$\begin{array}{c} \text{μAmp}|\text{Arousa}l_{\left[i\right]}=\mathrm{\mu Am}p_{\left[\mathrm{Par}\left[i\right]\right]}\\ +\frac{\text{σAmpAr}o_{\left[\mathrm{Par}\left[i\right]\right]}}{\mathrm{\sigma Ar}o_{\left[\mathrm{Par}\left[i\right]\right]}^{2}}\left(\text{Arousa}l_{\left[i\right]}-\mathrm{\mu Ar}o_{\left[\mathrm{Par}\left[i\right]\right]}\right) \end{array}$$

(22) $$\text{σAmp}|\text{Arousa}l_{\left[i\right]}^{2}=\mathrm{\sigma Am}p_{\left[\mathrm{Par}\left[i\right]\right]}^{2}-\frac{\text{σAmpAr}o_{\left[\mathrm{Par}\left[i\right]\right]}}{\mathrm{\sigma Ar}o_{\left[\mathrm{Par}\left[i\right]\right]}^{2}}$$

Summary statistics comparing the models for both the scene‐LPP and video oscillation‐power can be found in Table 1. The models were compared in terms of per‐trial variance explained (*R* ^2^) by the full model and when only looking at the predictor of interest (stimuli for Model 1 and arousal rating for Model 2). The posterior for *R* ^2^ is defined here through the following equation:

(23) $$R^{2}=\frac{\text{variance of predicted observations}}{\left(\text{variance of predicted observations}+\text{residual variance}\right)}$$

**TABLE 1:** Arousal model better explains video‐power than scene‐LPP: Bayesian Multilevel Model summary statistics.

Models	*R* ^2^ [95% CI]	Predictor‐*R* ^2^ [95% CI]	ELPD (SE)	Weight
Scene‐LPP				
Model 1: Stimuli Predictor	0.150 [0.129, 0.172]	0.068 [0.052, 0.086]	0.00	0.92
Model 2: Arousal Predictor	0.090 [0.072, 0.110]	0.018 [0.009, 0.028]	−102.3 (15.6)	0.08
Video‐power				
Model 1: Stimuli Predictor	0.687 [0.674, 0.698]	0.042 [0.030, 0.055]	0.00	0.76
Model 2: Arousal Predictor	0.669 [0.656, 0.681]	0.035 [0.023, 0.048]	−59.9 (15.6)	0.24

*Note:* Two models were fit to the single‐trial data for the scene‐LPP and video oscillation‐power. Here summary statistics report how much variance was explained (*R* ^2^ posteriors) for each full model and just the predictor (stimuli or arousal rating) minus the effect of participant. More important is the metric of cross‐validation that penalizes overfit models. For Bayesian models, cross‐validation accuracy was measured by expected log point density (ELPD) via the PSIS‐LOO method. A larger ELPD indicates the model would be more predictive for future data. Cross‐validation accuracy can also be interpreted by model weights. The weights indicate what linear combination of the models would be the most predictive in terms of cross‐validation accuracy.

This gives a posterior density for *R* ^2^ because there are posteriors for each predicted observation. The predictor *R* ^2^ was found by removing the predicted effect for each participant via subtracting these predictions from the observed data. Then the same variance explained equation was used to find this predictor specific *R* ^2^.

Cross‐validation was assessed with the PSIS‐LOO method (Vehtari, Gelman, and Gabry 2017). Generally, this method finds cross‐validation accuracy by weighting the likelihood of each observation by how much the model would change if the observation was excluded from model fitting. The *k* diagnostic statistics were within an acceptable range indicating the cross‐validation accuracy was not affected by outliers and would lead to equivalent conclusions as other cross‐validation algorithms or information criteria (Vehtari, Gelman, and Gabry 2017). Cross‐validation is reported in the units of expected log‐predictive density (ELPD) with more positive values indicating better accuracy. The difference in ELPD between models is reported in Table 1. Cross‐validation accuracy was also used to find stacking weights, which can aid interpretation. The weights indicate which model had more cross‐validation accuracy and what linear combination of the models would lead to the best cross‐validation accuracy. For example, if Models 1 and 2 were weighted 0.6 and 0.4 respectively, then Model 1 is the “best” model, but the most predictive guess for future data would use 60% of Model 1 with 40% from Model 2.

## 3 Results

### 3.1 Group‐Level Statistics

**FIGURE 2:**
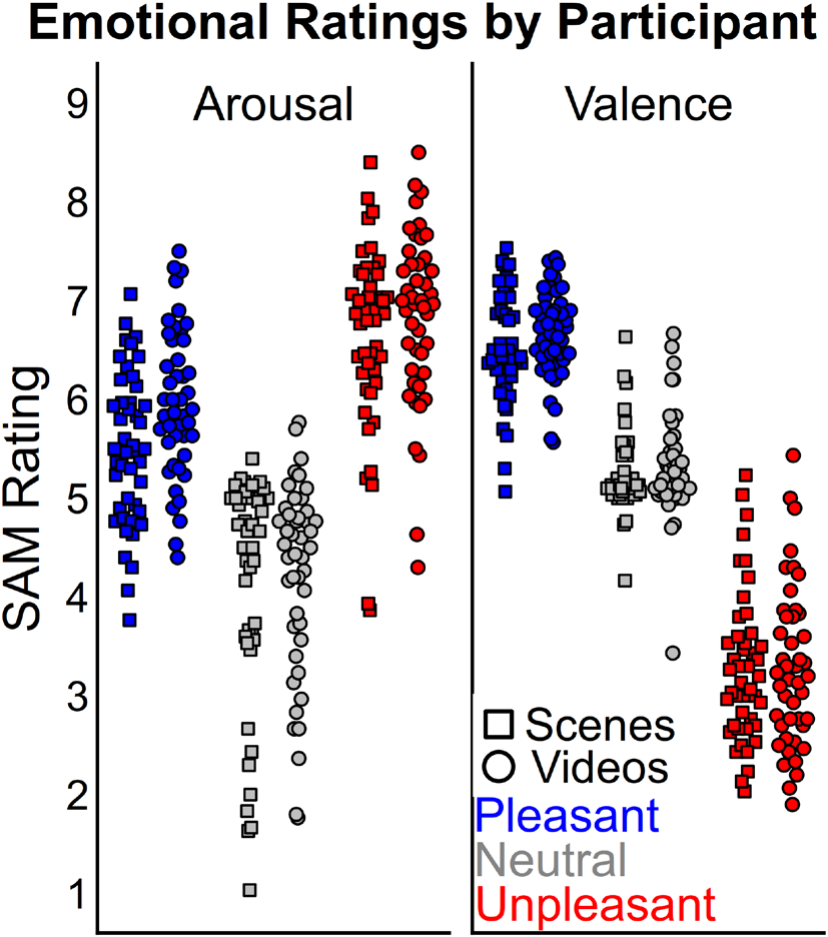
Valence and Arousal ratings for the three predetermined categories of pleasant, neutral, and unpleasant content. The complete scale from 1 to 9 is shown here. Each category featured 30 stimuli for 90 stimuli total. Pleasant and unpleasant videos were rated more arousing than scenes. The stimuli did not differ in valence ratings.

**FIGURE 3:**
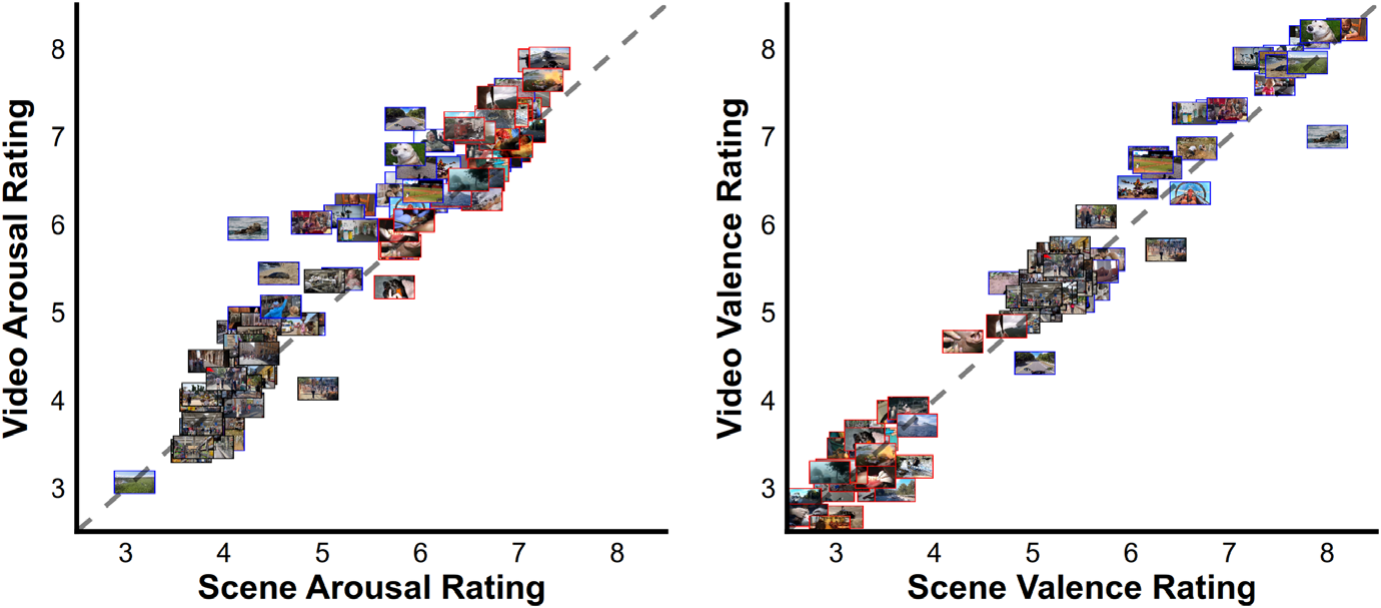
Average video and scene arousal ratings represented by the scene that was presented. All but 5 (of 90) of the scenes were selected from the most representative frame of the corresponding video. The colored borders around each scene indicate if the scene belonged to the pleasant (blue), neutral (black), or unpleasant (red). More pleasant and unpleasant videos were rated as more arousing than their scene matched pairs. Valence ratings between the videos and scenes were not statistically different.

For SAM emotion ratings, there were statistical differences for valence and arousal ratings between scenes and videos (Figures 2 and 3). The first factorial ANOVA on arousal ratings found a significant interaction (*F* (2, 88) = 8.69, *p* < 0.001; *η*_p_^2^ = 0.16) between stimulus type (scenes or videos) and emotional category (pleasant, neutral, or unpleasant). Breaking this down shows that arousal ratings were significantly different for scenes (*F* (2, 88) = 82.89, *p* < 0.001; *η*_p_^2^ = 0.65) and videos (*F* (2, 88) = 117.48, *p* < 0.001; *η*_p_^2^ = 0.73). Paired *t*‐tests found that pleasant videos (*M* = 5.98, SE = 0.12) were more arousing than pleasant scenes (*M* = 5.54, SE = 0.12; *t*(44) = 5.5, *p* < 0.001; *D* = 0.83) and unpleasant videos (*M* = 6.81, SE = 0.13) were more arousing than unpleasant scenes (*M* = 6.63, SE = 0.14; *t* (44) = 3.16, *p* = 0.003; *D* = 0.47). Neutral videos (*M* = 4.22, SE = 0.15) were not rated differently in arousal compared with neutral scenes (*M* = 4.15, SE = 0.18; *t* (44) = 0.47, *p* = 0.469; *D* = 0.11). For valence ratings, there was not an interaction between stimuli type and valence category; *F* (2, 88) = 1.43, *p* = 0.245; *η*_*p*_^*2*^ = 0.03. There was a significant effect of valence for scenes (*F* (2, 88) = 288.03, *p* < 0.001; *η*_*p*_^*2*^ = 0.87) and videos (*F* (2, 88) = 347.52, *p* < 0.001; *η*_p_^2^ = 0.89). For the valence ratings of scenes, pleasant scenes received an average rating of 6.57 (SE = 0.09), neutral scenes received 5.23 (SE = 0.06), and unpleasant scenes received 3.31 (SE = 0.12). Pleasant videos received a rating of 6.69 (SE = 0.08), neutral videos were rated as 5.32 (SE = 0.08), and unpleasant videos at 3.31 (SE = 0.12).

**FIGURE 4:**
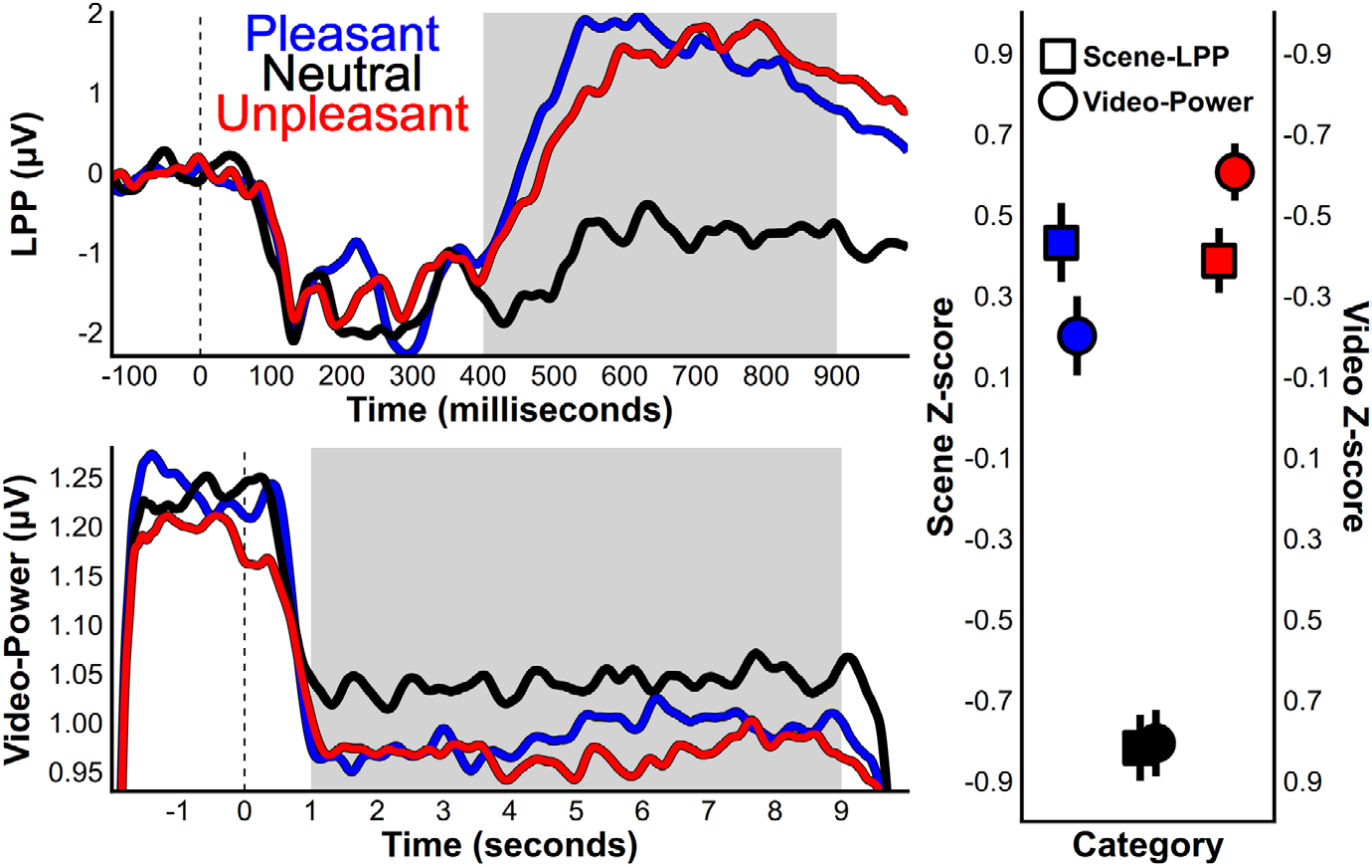
Averaged waveforms for the scene LPP (top) and video oscillation‐power (bottom). For the group category analyses, both measures were Z‐scored within participant. Z‐scored means and standard errors are depicted in the plot at the right. Typical LPP modulation was found such that pleasant and unpleasant content evoked more positive voltage over central‐parietal sensors. For videos, at 0 s the scrambled video and audio baseline transitioned into one of the 90 videos over 0.5 s or 12 frames. The sharp drop in amplitude seen at this latency supports the idea that attentional engagement is indexed by oscillation‐power amplitude as the video become coherent. The oscillation‐power amplitude decrease was stronger for emotional content, and the effect of emotion was not statistically different from the Z‐scored LPP.

As with the ratings, ANOVAs and *t*‐tests found significant differences for the by‐category Z‐scored amplitudes for the scene‐LPP and video oscillation‐power (Figure 4). A factorial ANOVA did not find a significant interaction between stimulus type and valence category (*F* (2, 88) = 2.20, *p* = 0.116; *η*_p_^2^ = 0.05). There was a significant effect for valence category for the scene‐LPP (*F* (2, 88) = 44.39, *p* < 0.001; *η*_p_^2^ = 0.50) and oscillation‐power (*F* (2, 88) = 49.37, *p* < 0.001; *η*_p_^2^ = 0.53). The Z‐scored mean for pleasant scenes was 0.43 (SE = 0.10), neutral was −0.82 (SE = 0.08), and unpleasant was 0.39 (SE = 0.08). For videos, the pleasant Z‐scored amplitude was 0.20 (SE = 0.10), neutral was −0.81 (SE = 0.08), and unpleasant was 0.61 (SE = 0.07).

**FIGURE 5:**
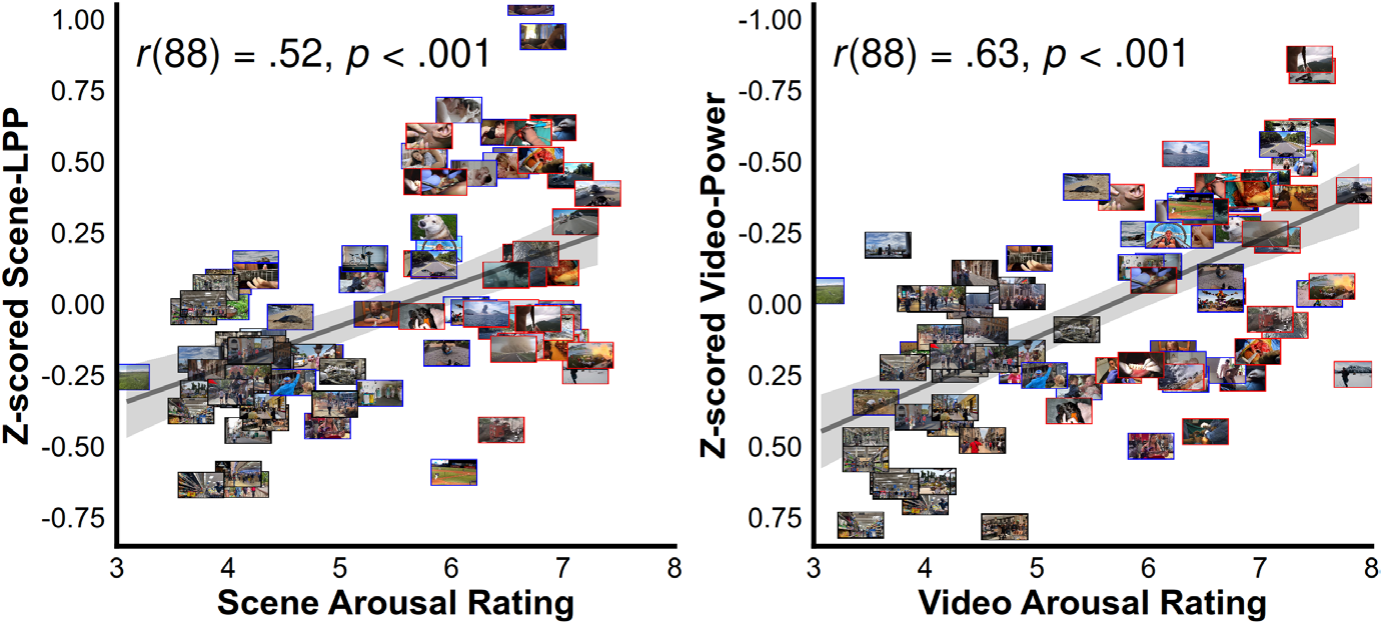
Correlations of the average arousal rating and Z‐scored scene‐LPP (left) and video oscillation‐power (right) amplitudes are plotted for the 90 scene and video stimuli. For visual clarity of the figure, the y‐axis was reversed for the video plot. Both EEG measures were strongly correlated with arousal ratings. Consistent with the literature, there appears to be a larger LPP response for erotic and gory content, whereas oscillation‐power does not appear to show such a sensitivity. This point is expanded upon in the text and with subsequent multilevel Bayesian analyses.

Average arousal ratings per scene and video were correlated with the average Z‐scored amplitude (Figure 5). When amplitudes per stimuli were Z‐scored within each participant and then averaged, arousal ratings were significantly correlated with LPP amplitude (*r* = 0.52, *p* < 0.001) and video oscillation‐power (*r* = −63, *p* < 0.001). The interaction via a linear model between arousal ratings and EEG measure (scene‐LPP or oscillation‐power) was not significant (*F* (1, 176) = 0.45, *p* = 0.50; *η*_p_^2^ = 0.003).

### 3.2 By‐Trial Multilevel Bayesian Analyses

**FIGURE 6:**
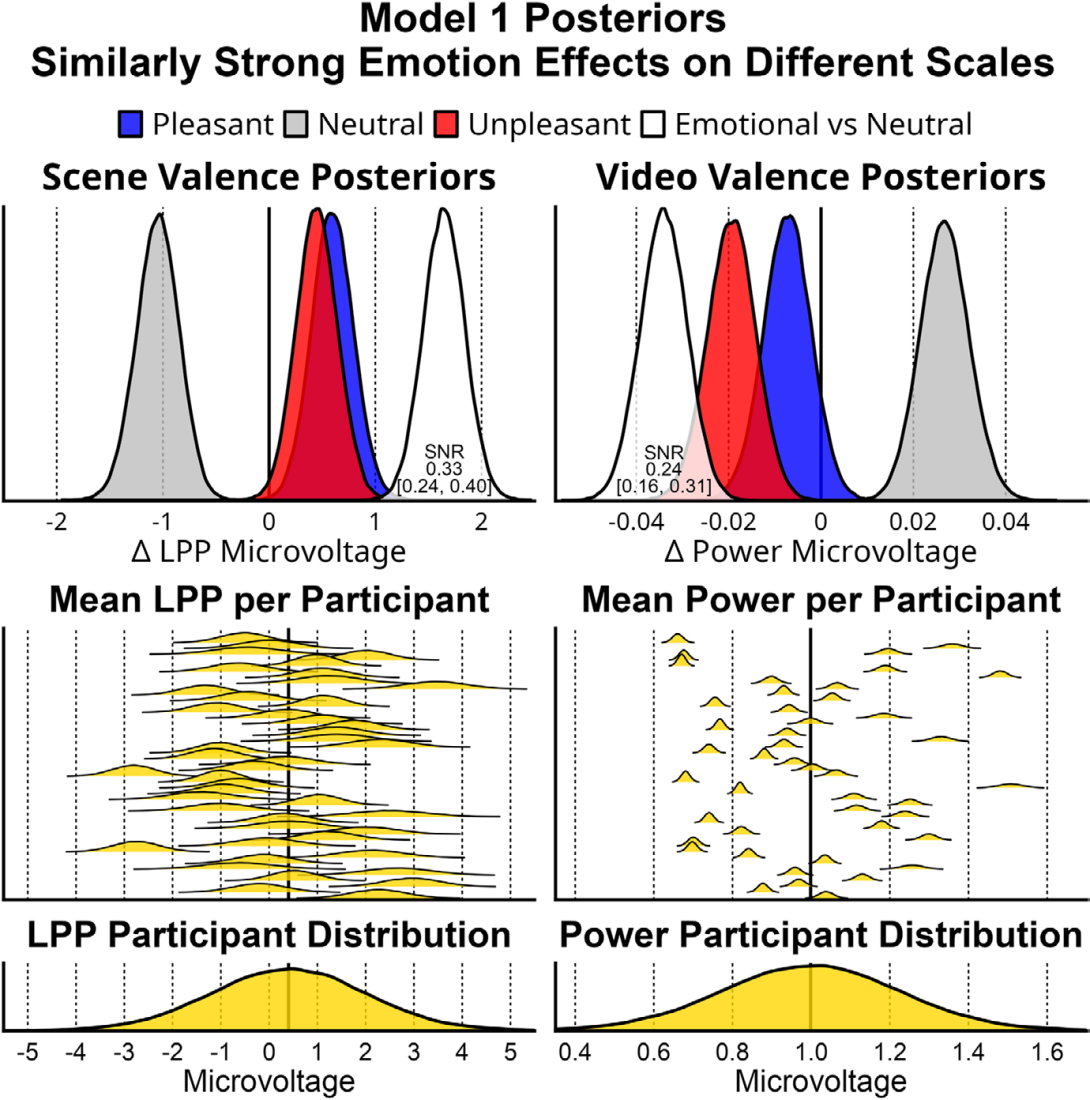
Depicted are the relevant posteriors estimated for Model 1 that predicts the cortical amplitudes based on stimulus content. The top row depicts the average posteriors (*Beta2*) for pleasant, neutral, and unpleasant content as well as the difference between emotional versus neutral. The second row are the estimated participant means (*Beta1*). The third row is the estimated distribution of participant means (Normal(*ParBar*, *sigmaPar*)). Despite different scales for the measurements and between‐participant effects, there appears to be strong evidence for a consistent emotional category effect evident by the emotional difference (white curve) not containing zero. The complete set of posteriors is a generative model helpful for replication efforts, power‐analyses, and meta‐analyses.

Summary statistics for each of the Bayesian multilevel models can be found in Table 1. The full models for video oscillation‐power explained more variance (*R* ^2^) because of larger between‐subject effects for this cortical measure. This is evident in the larger distance in estimated participant mean posteriors (*Beta1*) in Figure 6. For the scene‐LPP, Model 1 that used the 90 stimuli as predictors explained more variance than Model 2 that used arousal ratings. For oscillation‐power, the *R* ^*2*^ posteriors for models overlapped. The amount of variance explained by stimuli or arousal ratings alone (Predictor‐*R* ^2^) can be considered a statistic to gauge a per‐trial effect size or SNR for those predictors. For the LPP, stimuli predicted more single trial variance than arousal ratings; median = 0.051 [0.032, 0.071]. For oscillation‐power, the stimuli and arousal predictors explained statistically equivalent amounts of variance; median = 0.007 [−0.011, 0.025]. Cross‐validation accuracy indicates that Model 1, that used the 90 stimuli as predictors, would be best able to forecast future data for the scene‐LPP and oscillation‐power. Model weights reflect how model predictions could be best combined to forecast future data. In Table 1, the weights indicate that the scene‐LPP would best be forecast by the stimuli‐based Model 1 primarily (92% of prediction). For oscillation‐power, Model 1 was also most predictive (76%), though the arousal rating‐based Model 2 was more predictive (24%) than it was for the LPP (8%).

The relevant Model 1 posteriors are depicted in Figure 1, and the complete set of estimated parameters can be found in the supplemental materials and on the project's open science framework page (https://osf.io/gx243/). For the scene‐LPP stimuli posteriors, the averaged pleasant‐content samples estimated a posterior with a median of 0.60 μV [0.21, 0.99], neutral content led to −1.05 μV [−1.45, −0.66], and unpleasant content led to 0.45 μV [0.07, 0.84]. For the ssVEP, the pleasant‐content posterior had a median value of −0.007 μV [−0.018, 0.003], neutral content led to 0.027 μV [0.017, 0.037], and unpleasant content led to −0.019 μV [−0.030, −0.009]. The posterior for the emotional difference from neutral found support for both EEG measures, as neither density contained zero; LPP median = 1.65 μV [1.26, 2.03] and video oscillation‐power median = −0.034 μV [−0.044, −0.024]. For the LPP the per‐trial signal to noise ratio for the difference between emotional and neutral stimuli was 0.33 [0.24, 0.40], and 0.24 [0.16, 0.31] for oscillation‐power. A posterior contrast of these SNR posteriors was not statistically significant at our default 95% credibility interval; median 0.09 [−0.02, 0.20].

**FIGURE 7:**
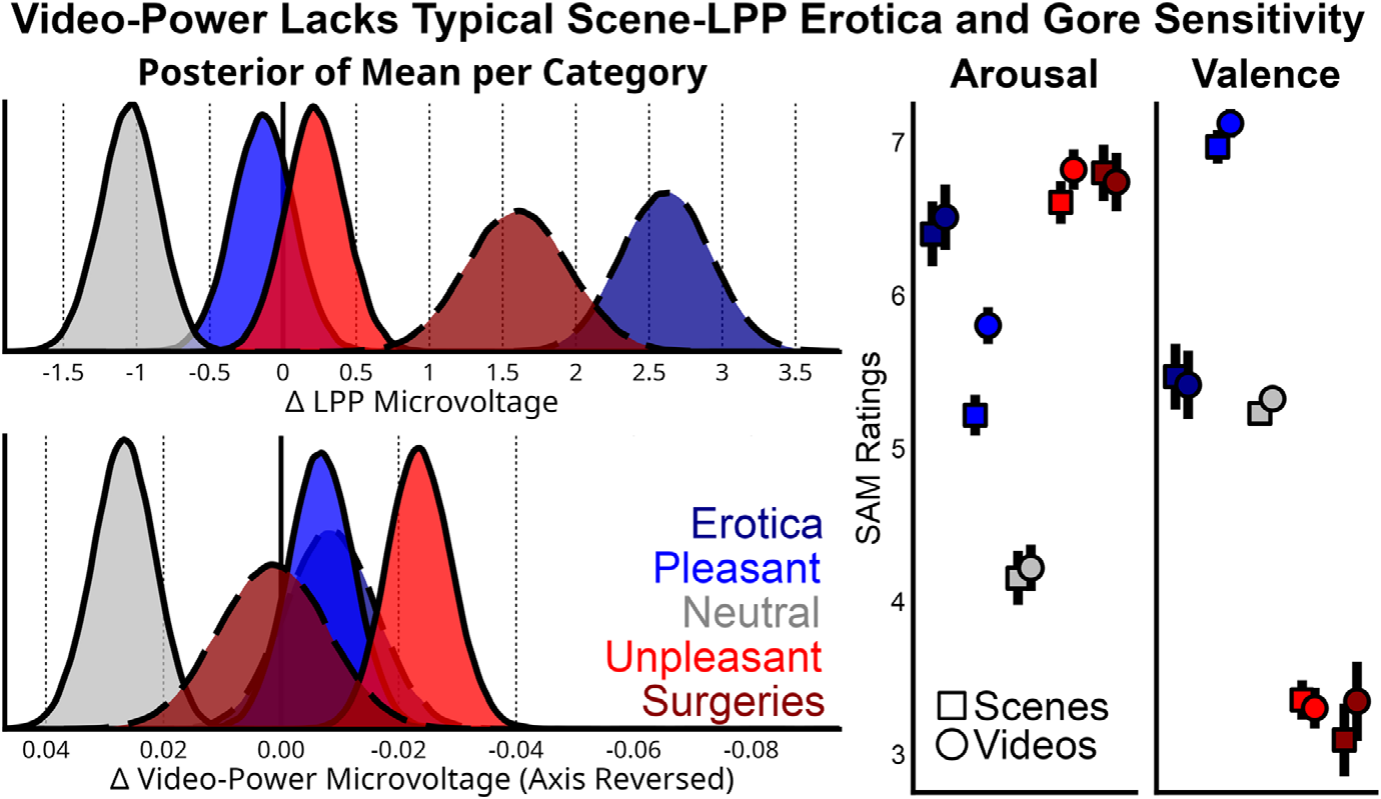
Depicted are the same posterior means from Model 1 rearranged to separate stimuli that depicted erotica and surgeries, as well as the average SAM ratings with standard error bars to contextualize the posteriors. It is commonly reported that erotica and mutilation content have an outsized effect on the LPP. Here the Bayesian model is well‐positioned to provide post hoc comparisons by separating out the posteriors of the 8 erotic couples and 5 surgery stimuli from the remaining pleasant and unpleasant posteriors. As described in the methods, mutilation videos are difficult to gather, so surgery videos were used that also feature gory depictions. For the LPP, there was strong evidence that these specific types of content produced greater amplitudes. The video oscillation‐power did not show this sensitivity. The remaining pleasant and unpleasant posteriors indicate more statistical certainty of an emotional effect for oscillation‐power compared to the scene‐LPP relative to neutral content.

The Model 1 Stimuli posteriors rearranged to understand if erotic and gory content differential modulated the scene‐LPP versus the oscillation‐power (Figure 7). For the LPP, the median sample of the erotica posterior was 2.62 μV [2.03, 3.23], pleasant (nonerotic) led to −0.14 μV [−0.55, 0.28], neutral led to an unaltered −1.05 μV [−1.45, −0.66], unpleasant was 0.22 μV [−0.18, 0.63], and surgeries led to 1.60 μV [0.93, 2.29]. For oscillation‐power, erotica led to −0.008 μV [−0.024, 0.007], pleasant (nonerotic) led to −0.007 μV [−0.018, 0.004], neutral led to 0.027 μV [0.017, 0.037], and unpleasant led to −0.024 μV [−0.035, −0.013], while mutilation content led to 0.001 μV [−0.017, 0.020]. To contextualize these posteriors, we plot the valence and arousal ratings in Figure 7 also split by these 5 post hoc categories. Erotic scenes received an average arousal rating of 6.39 (SE = 0.21) & valence rating of 5.47 (0.21), pleasant 5.21 (0.13) & 6.95 (0.11), neutral 4.16 (0.17) & 5.23 (0.06), unpleasant 6.58 (0.14) & 3.37 (0.13), and surgeries 6.73 (0.19) & 3.09 (0.23). Erotic videos had an average arousal rating of 6.51 (SE = 0.21) & valence rating of 5.43 (0.22), pleasant 5.80 (0.12) & 7.11 (0.09), neutral 4.22 (0.15) & 5.32 (0.08), unpleasant 6.80 (0.13) & 3.31 (0.13), and surgeries 6.73 (0.19) & 3.33 (0.25).

Model 2 found that arousal ratings were a better predictor of the video oscillation‐power by trial. The overall correlation between arousal ratings and amplitude was larger for the video‐power (*r* = −0.16, [−0.21, −0.11]) than for the LPP (*r* = 0.10, [0.06, 0.14]). A posterior contrast found that there is a 96% probability that the correlation is larger for video oscillation‐power compared wit the scene‐LPP. For the regularized by‐participant correlations, there were more statistically meaningful correlations for oscillation‐power (21 out of 45 participants) versus the scene‐LPP (13 participants) evident through 95% credibility intervals. The factors of participant sex, age, racial/ethnic identity, nor amplitude or arousal variability was related to correlation strength. However, for readers interested in individual differences, the complete set of posteriors per participant can be found in the supplemental materials.

### 3.3 Fourier Transform Video Power Analysis

**FIGURE 8:**
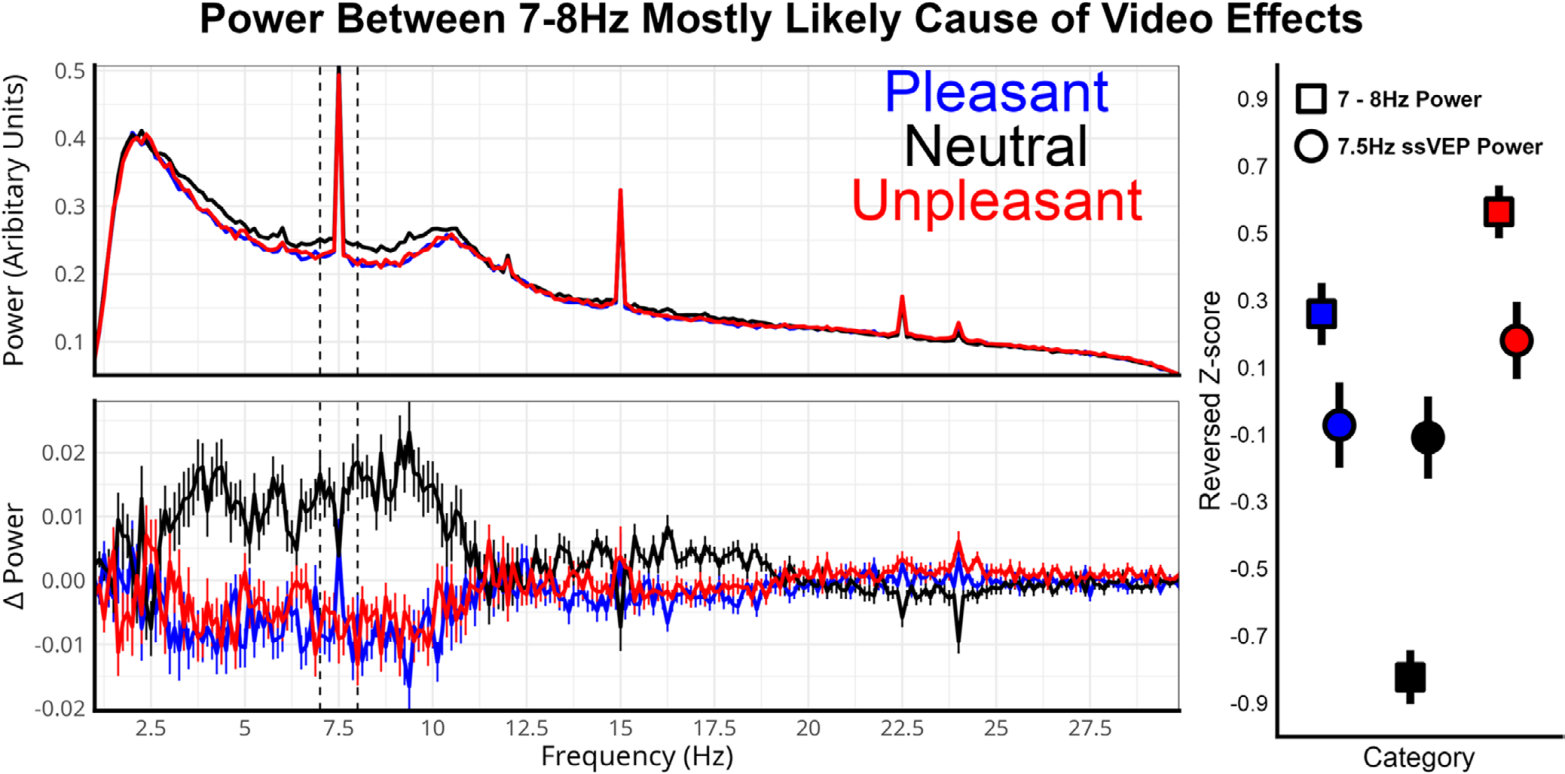
Fourier Transform analysis of the occipital sensors during video presentation per valence category. Top‐left is the power per each frequency split by valence category. Frequencies at 3 Hz and lower get attenuated by high‐pass filter. The ssVEP driving frequency is 7.5 Hz with harmonics at 15 and 22.5. All videos were presented at 24 frames per second on a 60 Hz monitor. At bottom‐left is the mean power per valence category when subtracted from the mean within each participant. Standard errors are represented as bars at each frequency. The right plot is the power Z‐scored by category between 7 and 8 Hz (dotted lines in left plots) and the 7.5 Hz ssVEP frequency. Statistics for the 7–8 Hz power are near equivalent to narrow‐band Hilbert measures that use filtering cut‐offs of 7 to 8 Hz. Power at the 7.5 Hz ssVEP frequency did not statistically differ based on category.

During peer review, it was discovered that inherent oscillation changes including the 7–8 Hz window were the most likely cause of video‐power effects. This is shown with a Fourier Transform of the same video EEG data using the same epoch and occipital channels (Figure 8). The spectrum results show that there is a reliable drop in power from 3 to 10 Hz. A repeated measure ANOVA of Z‐scored power between 7 and 8 Hz was statistically significant with similar *F*‐value and effect size as Hilbert‐derived power; *F* (2, 88) = 49.84, *p* < 0.001; *η*_p_^2^ = 0.53. Whereas, the same analysis of the driving ssVEP frequency of 7.5 Hz was not statistically significant; *F* (2, 88) = 1.12, *p* = 0.332; *η*_p_^2^ = 0.03. Z‐scored amplitudes for power between 7 and 8 Hz were very similar to Hilbert‐derived power; pleasant = −0.259 (SE = 0.09), neutral = 0.823 (SE = 0.08), and unpleasant = −0.564 (SE = 0.8). The same analysis on 3–10 Hz power (selected post hoc) found a larger effect between categories; *F* (2, 88) = 112.17, *p* < 0.001; *η*_p_^2^ = 0.72. For this frequency window, the Z‐scored pleasant mean was −0.63 (SE = 0.05), neutral 0.96 (0.06), and unpleasant was −0.33 (SE = 0.08). Wavelets of power are depicted in the supplemental materials.

## 4 Discussion

The present study assessed the extent to which emotional brain reactivity can be measured with ssVEP during realistic multimodal video perception, as compared to the widely employed modulation of the LPP to emotional scene stimuli. To address this question, a single group of participants were presented with 90 matched pairs of videos and scenes. The self‐reported emotion ratings show that emotional videos were rated as slightly more arousing than scenes, while valence ratings were not statistical different (Figures 2 and 3). Initial analyses suggested that the video‐ssVEP had a comparable emotion effect to the scene‐LPP, with the added benefit of being more strongly associated with individual arousal ratings allowing for a better assessment of emotion from a broader range of content. However, during peer review, it was discovered that the video emotion effect was in fact driven by a wider drop in power that included 7–8 Hz, while the emotion effect at 7.5 Hz precisely was not significant (Figure 8). For this reason, the Hilbert‐derived video measure is now more accurately referred to as video oscillation‐power instead of ssVEP power. In light of this, the scene‐LPP may be considered a better marker of emotional arousal than the video‐ssVEP as originally implemented. However, the large emotion‐related differences in video oscillation‐power suggest that well‐controlled emotional videos may provide a reliable metric of evoked emotional states using video‐ and participant‐specific analysis methods, though this adds complexity to the interpretation of underlying mechanisms as compared to the ssVEP approach.

As described here and in previous research (Sabatinelli, Farkas, and Gehr 2024), emotion video research comes with significant challenges because of the complexity of measuring brain activity evoke by dynamic‐multisensory information. We address this issue with standardized videos and ssVEPs that is theoretically a robust measure of stimulus engagement that is reliable across videos and participants. Although we evoked a reliable ssVEP at the driven frequency of 7.5 Hz, confirming precise timing during the experiment (Figure 8), there was not an emotional effect at that specific frequency. Previous studies have shown that scenes and classically conditioned stimuli can reliably alter a competing ssVEP response (Wieser, Miskovic, and Keil 2016), but a common element is that the competing stimuli often spatially overlap. It has been stated that overlap may be an essential element to reliably assess emotion, so future video paradigms may either need to overlay flickering dots or Gabor‐patches on videos, despite potential reductions in ecological validity (Müller, Andersen, and Keil 2008). Other options for improving the ssVEP paradigm would be to flicker the video itself, which has been done in previous research (Stegmann et al. 2023).

The results suggest that standardized videos evoke reliable emotional differences across a range of low frequencies, not limited to the conical alpha‐band (8–12 Hz). This could be the basis of further improving measures of emotion during video viewing without the need of a ssVEP‐inducing flicker, but such frequency analyses considerably increase the complexity of interpretation. While oscillation frequency analysis in EEG is common and used to assess emotions in video research, this is typically done on prespecified frequency bands such as alpha from 8 to 12 Hz (Romeo et al. 2022; Romeo, Angrilli, and Spironelli 2024). The present data suggest that we have large effect sizes despite analyzing a small portion of the frequency spectrum, with the effect getting larger when the data is analyzed from 3 to 10 Hz. Instead of a shift in a specific frequency bands, a visualization of a wider range of frequencies suggests this large difference is due to a shift in background power termed scale‐free (He 2014) or aperiodic power that appears as a power spectrum with a 1/frequency shape (1/f^β). Measuring the shifts in the aperiodic power is becoming increasingly common as algorithms have been introduced to quantify aperiodic and oscillatory bands changes simultaneously (Donoghue et al. 2020). Yet it has been suggested that the general 1/f^β shape can hide considerable computational complexity that is still being understood. There can be “knees” in the 1/f^β shape in which the exponent can change at specific frequencies (Miller et al. 2009), the phase of lower frequencies may modulate the power of higher frequencies (He et al. 2010), the 1/f^β differs based on scalp position (He 2014), or the 1/f^β can differ between imaging methods (e.g., ECoG, EEG, or MEG; He 2014). Certainly, forming a reliable description and interpretation of 1/f^β and frequency‐band emotional video modulation is beyond the scope of this manuscript, and will require additional data as well as targeted modeling and analysis.

The LPP and the emotion‐modulated early posterior negativity (EPN) have primarily been discussed through a theory of “motivated attention,” in which emotional content inherently evokes selective attention toward a scene (Bradley 2009; Ferrari et al. 2008; Hajcak, Dunning, and Foti 2009; Olofsson et al. 2008; Schupp et al. 2000, 2004). A portion of this modulation is likely due to emotion related attention, as more emotionally intense depictions of similar content increase the LPP, consistent with arousal ratings and skin conductance (Cuthbert et al. 2000). Moreover, identical scenes can evoke larger or smaller LPP amplitudes based on framing or emotion regulation (Hajcak, MacNamara, and Olvet 2010; MacNamara, Joyner, and Klawohn 2022). However, habituation studies challenge the idea that emotion modulated ERPs purely reflect experienced emotion and attention. Large reliable ERP emotional differences still occur even when a small number of scenes are presented hundreds of times, which abolishes emotional differences for other physiological measures such as skin conductance and pupil dilation (Codispoti, Ferrari, and Bradley 2006, 2007; Ferrari et al. 2011, 2016; Ferrari, Mastria, and Codispoti 2020; Mastria, Ferrari, and Codispoti 2017). Separately, scenes that depict features commonly associated with emotional content (such as exposed bodies) can produce large LPP and EPN amplitudes, even though these scenes are rated as emotionally neutral (Farkas, Oliver, and Sabatinelli 2020; Farkas and Sabatinelli 2023). Taken together, these findings suggest that a considerable portion of scene‐evoked ERPs reflect obligatory processes of emotional feature discrimination that *may*, but need not necessarily facilitate attention or other emotional processes. This adjusted perspective could have important interpretive implications. Despite single and repeated scene paradigms both being able to elicit emotional ERP modulation, repeating trials may habituate the portion of the LPP related to emotional arousal. For research on individual differences and psychiatric disorders, group differences in the LPP may, for example, primarily reflect visual features differentially encoded as emotional rather than differences in attention or experienced emotion.

Bayesian multilevel models provided many benefits to the present study, thus highlighting the usefulness of the approach. The goal of most statistical analyses is to explain and predict a phenomena as well as possible while avoiding issues such as false‐positives, underfitting, and overfitting. Multilevel Bayesian models are growing in popularity because they often best balance all of these competing interests. By default, estimates are regularized and corrected for multiple comparisons because individual estimates are informed and constrained by the group estimate (Gelman, Hill, and Yajima 2012). In contrast, for non‐Bayesian methods, maximizing cross‐validation accuracy or correcting for spurious relationships are usually separate processes with numerous possible solutions. When statistical power and overfitting can be simultaneously controlled, the complexity and specificity of modeled phenomena can be improved. For example, estimating the effect for each individual stimuli (Model 1) or the correlation for arousal and amplitude per participant (Model 2) would usually not be justified because it would result in too many statistical tests as well as presenting an unrealistic interpretation caused by an overfit to the data available. Yet, these complex models outcompete simpler models in the most important metric of cross‐validation accuracy indicating there is not overfitting. This is critical as fields like neuroimaging and psychophysiology try to translate group‐level understanding into metrics for understanding individual differences or precise diagnostic information.

The present study was designed to provide a direct comparison between the scene evoked LPP and video modulated ssVEP, but not all factors could be completely balanced. For both stimuli types, the same number of trials and content were presented. However, because of the long video duration and inter trial interval, the video paradigm was longer and slightly more trials were contaminated by artifacts and were removed from final analyses (3441 scene versus 3358 video trials). Separately, the amount of scene trials could have been tripled such that both had an equal paradigm duration. This would likely have made category and content averaged results seem more favorable for the LPP measure. Future research teams will have to weigh their research priorities to decide whether the increased experiment duration and neurophysiological complexity is worth the use of videos.

Presenting scrambled video and audio before the actual video onset was informative to our interpretation of the results, but did not improve the accuracy of the ssVEP or video oscillation‐power measures. Prior to data collection, it was expected that the baseline period could be used to baseline‐correct each trial as is typically done with ERPs. However, when the baseline was subtracted, the average differences remained the same while the variance increased, thus lowering statistical power. This is because the 2 s of baseline period has its own random voltage fluctuations. Thus, those residual variances compound because there are two sources of error, during the baseline epoch and during the video presentation. Thus, future use of this paradigm need not include the scrambled baseline period, so the procedure could be shortened to include more trials.

## 5 Conclusion

Recent work found that short, realistic video clips differing in emotional content prompt large arousal‐related differences in ssVEP amplitude. Here, we sought to replicate and extend this result by comparing matched videos to scenes that evoke the well‐established LPP index of emotion. In the process, we discovered that the ssVEP effect was driven by a portion of a broader drop in EEG power over a range of frequencies, and that no emotional effect was present at the precise ssVEP frequency. Further analyses of individual trials suggests that video oscillation‐power correlates strongly to rated arousal and appears to be less sensitive to erotic and gory content relative to LPP modulation. These results are intriguing because they suggests the two EEG measures index different aspects of emotional perception, both of which could be useful metrics in the study of emotion. The LPP may primarily reflect obligatory recognition processes of emotional versus neutral features that may be somewhat distinct from experienced emotion states. The LPP is thus especially useful for research questions about how cues may be differentially categorized between groups of people. EEG oscillatory power modulation during video perception appears to more strongly mirror reported emotional arousal. Future work in aperiodic modeling and traditional EEG frequency bands will be useful to develop a highly‐resolved measure of emotional engagement during videos that could in turn provide a powerful means to investigate various aspects of emotion, attention, and perception.

## Author Contributions

**Andrew H. Farkas:** conceptualization, data curation, formal analysis, investigation, methodology, project administration, software, supervision, validation, visualization, writing – original draft, writing – review and editing. **Matthew C. Gehr:** conceptualization, data curation, formal analysis, investigation, methodology, project administration, software, supervision, validation, visualization. **Han Jia:** formal analysis, software, validation, visualization, writing – review and editing. **Dean Sabatinelli:** conceptualization, funding acquisition, investigation, methodology, project administration, resources, supervision, validation, writing – review and editing.

## Conflicts of Interest

The authors declare no conflicts of interest.

## Supporting information


Data S1:

## Data Availability

The code and data that support the findings of this study are openly available at https://osf.io/gx243/.
