# Supplementary material for: Measuring Realistic Emotional Perception With EEG: A Comparison of Multimodal Videos and Naturalistic Scenes
Source: Psychophysiology. 2025 Jan 27;62(1):e14765. doi: 10.1111/psyp.14765 (PMC11771703; doi:10.1111/psyp.14765)
Supplement: Supplementary file 1 — Data S1: [file PSYP-62-e14765-s001.docx]

**Supplement**

By category group-level analyses were performed on amplitudes Z-scored within each participant enable comparison between the scene-LPP and video-ssVEP. Here we present the underlying data before and after Z-score scaling, as well as the difference in microvoltage from each participant’s mean. It is reasonable to ask if Z-scoring inflates the category effect, but it does not as repeated measure ANOVAs on raw microvoltage and Z-scored amplitudes lead to the same *F*-values and effect size measures of partial eta squared (*η_p_^2^*).


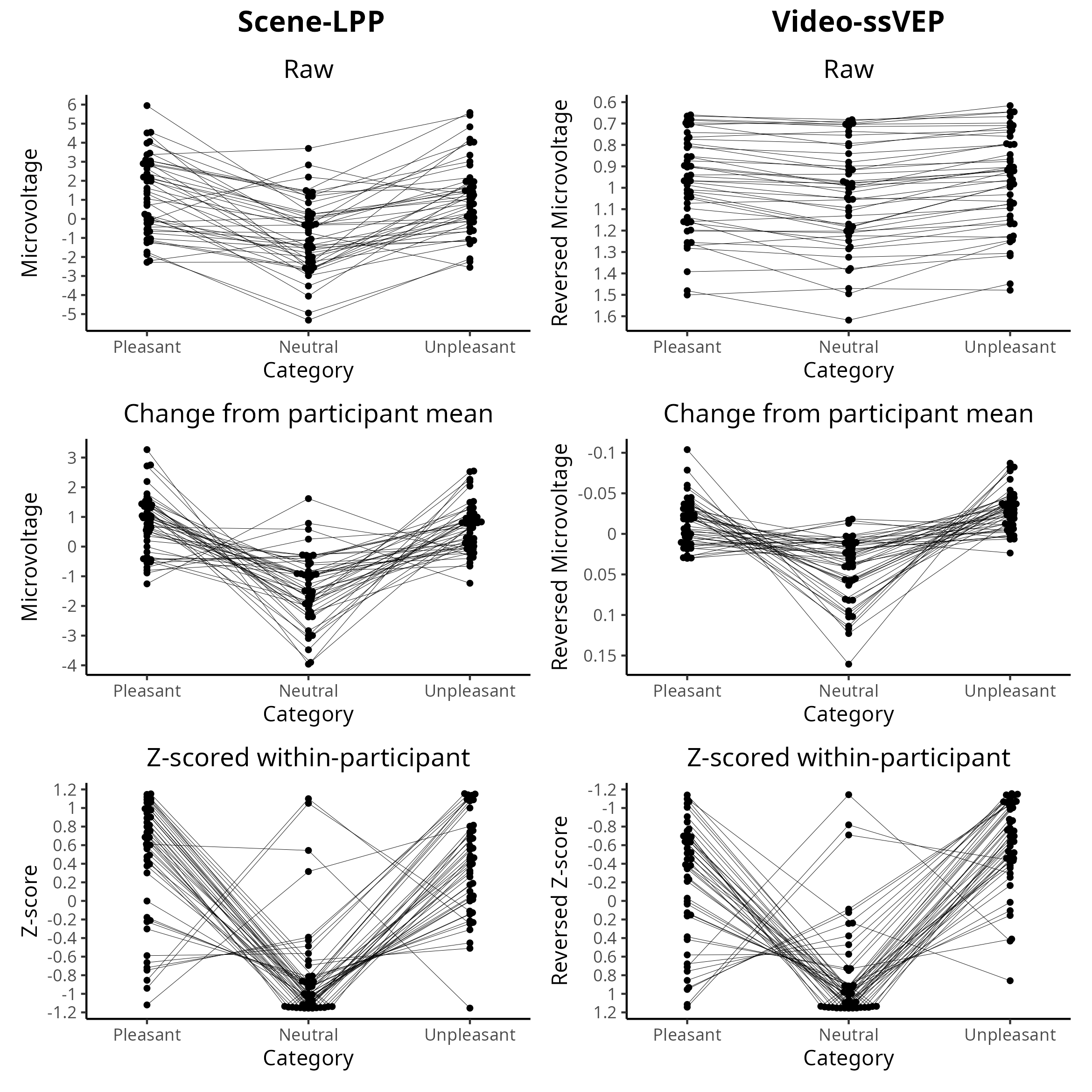


**Supplemental Figure 1.** The average by category results per participant. The category amplitude for each participant is represented by a dot and values that came from the same participant are connected via a line. The top row is the observed amplitude. The second row subtracted the mean of each participant such that the three categories now center zero for each participant. The third row shows the effects of Z-scoring within each participant on the raw amplitudes which enabled group-level comparisons across the measures.

There was an observed reduction across many frequencies during video viewing, with the reduction being larger for emotional content. The same occipital channels were used as other video time-frequency analyses (POz, O9/I1, O1, OI1h, Oz, Iz, O2, OI2h, O10/I2). This drop in frequencies is depicted here using a convultion of a family of complex wavelets using a morlet constant of 10. This was implemented using the gener_wav function on the csea-lab github repository (<https://raw.githubusercontent.com/csea-lab/csea-lab/refs/heads/master/Libas/4wavelettrans/gener_wav.m>). Overall power was baselined to depict the percent difference from 1611 to 48 ms before video onset. All videos were associated with reduction across many frequencies. Many of the reductions were larger for emotional relative to neutral videos. It is unclear if specific frequency bands are affected versus a general reduction in power. Future studies will attempt to model both types of power.


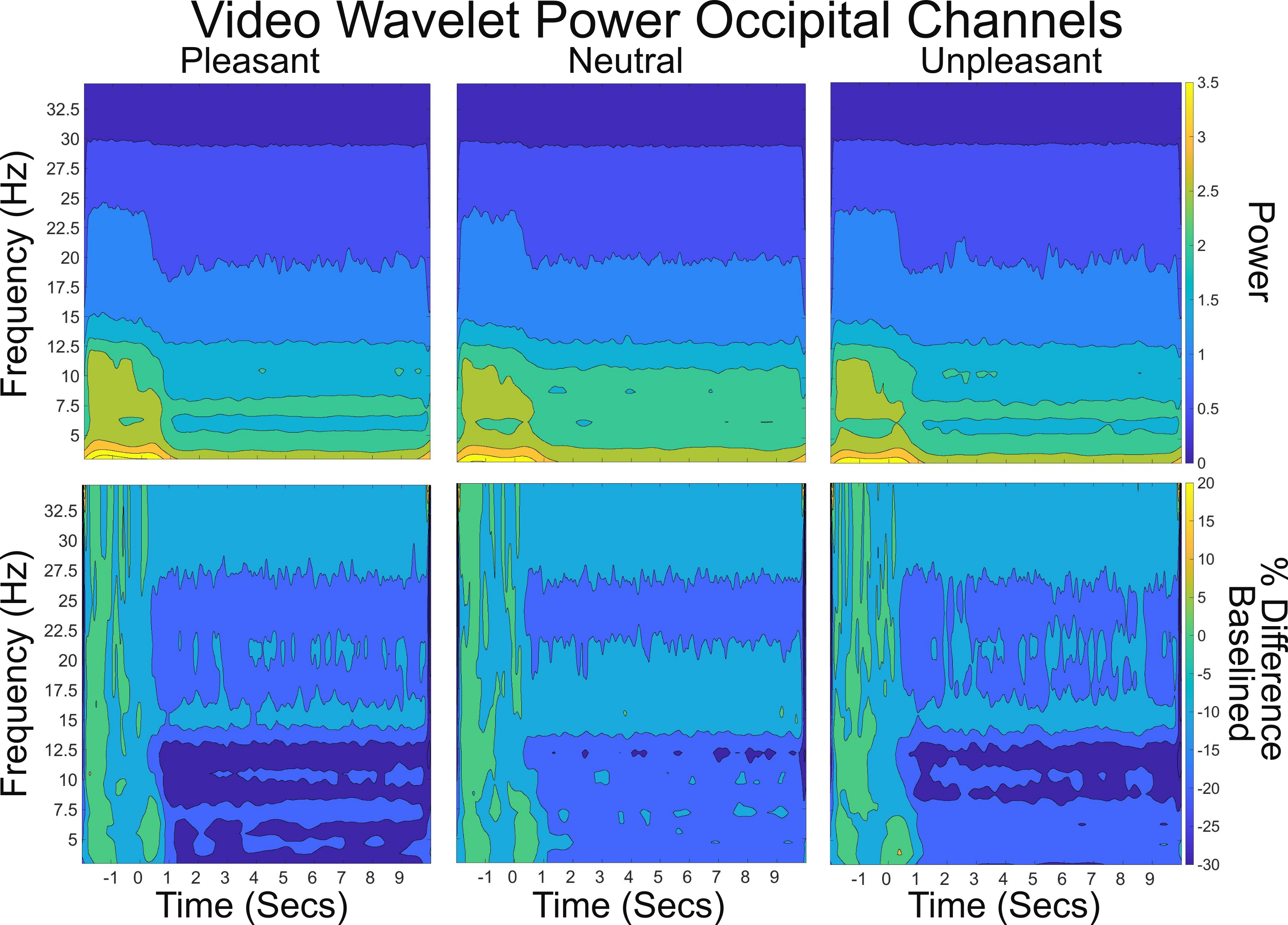


**Supplemental Figure 2.** Wavelet derived power realtive to video onset (zero on x-axis) over occipital EEG sensors. Top plots depict the power across frequencies over time. Bottom plots are the baseline percent difference from 1,611 milliseconds to 48 milliseconds before video onset.

Supplemental Table 1 displays the relevant data from Model 2 which predicted cortical amplitudes with arousal ratings. This addresses common questions and interests of readers, as well as showing how many participants had statistically meaningful correlations between electrocortical activity and arousal ratings. Readers commonly ask if results differed based on age, sex, and race/ethnicity; as well as other factors such as if participants used the rating scale differently. These types of analyses are usually underpowered and would lead to an inappropriate amount of false-positives due to multiple comparisons. Reporting tests of individual factors is also not incredibly meaningful as it seems likely there are complex intersectional relationships that need to be modeled in concert (Routen et al., 2023). This problem is somewhat addressed in Bayesian multilevel models because individual estimates are constrained by a hierarchal structure. This regularizes estimates and corrects for multiple comparisons allowing for more credible statistical inference of granular features (Gelman et al., 2012). Most individual differences are still likely underpowered, but the posteriors from this and the other models (https://osf.io/gx243/) can be used for future meta-analyses. This is because the posteriors can become the new prior distributions as more data is collected (Gelman, 2014). More participants had a statistically meaningful correlation (greater than 97.5% of samples above or below zero) between arousal ratings and the ssVEP (21 out of 45 participants) than the LPP (13 / 45). The magnitude of the per trial correlation is larger and it does not seem to be systematically related to age, sex, race/ethnicity, or the variability of the cortical measures and arousal ratings.

|  |  |  |  | **Model 2 Posterior Medians [95% Credibility Interval]** | | | | | | | | | | |
| --- | --- | --- | --- | --- | --- | --- | --- | --- | --- | --- | --- | --- | --- | --- |
|  |  |  |  | **LPP** | | | | |  | **ssVEP** | | | | |
|  | **Sex** | **Age** | **Ethnicity** | **Mean Amplitude** | **SD Amplitude** | **Mean Arousal** | **SD Arousal** | **Correlation** |  | **Mean Amplitude** | **SD Amplitude** | **Mean Arousal** | **SD Arousal** | **Correlation** |
| Overall Estimate |  |  |  | 0.42  [-0.08, 0.94] | 5.33  [4.98, 5.70] | 5.45  [5.26, 5.65] | 1.82  [1.66, 1.98] | **0.10**  **[0.06, 0.14]** |  | 1.00  [0.93, 1.07] | 0.15  [0.14, 0.17] | 5.67  [5.50, 5.85] | 1.94  [1.80, 2.07] | **-0.16**  **[-0.21, -0.11]** |
| Participant 1 | Male | 18 | White | 2.12  [0.94, 3.29] | 5.62  [4.87, 6.53] | 5.52  [5.14, 5.90] | 1.78  [1.53, 2.09] | 0.06  [-0.08, 0.19] |  | 1.04  [1, 1.08] | 0.17  [0.14, 0.20] | 5.65  [5.25, 6.05] | 1.79  [1.54, 2.12] | **-0.17**  **[-0.32, -0.01]** |
| Participant 2 | Female | 21 | Asian | -0.05  [-1.19, 1.09] | 5.07  [4.34, 6.00] | 5.99  [5.49, 6.5] | 2.28  [1.97, 2.68] | 0.08  [-0.05, 0.2] |  | 0.88  [0.86, 0.91] | 0.11  [0.10, 0.13] | 6.43  [5.99, 6.87] | 2.02  [1.74, 2.36] | **-0.20**  **[-0.37, -0.03]** |
| Participant 3 | Female | 19 | White | 2.93  [1.74, 4.08] | 5.78  [5.05, 6.68] | 5.65  [5.42, 5.87] | 1.07  [1.00, 1.23] | -0.01  [-0.21, 0.16] |  | 0.97  [0.93, 1.00] | 0.15  [0.13, 0.17] | 5.66  [5.40, 5.92] | 1.19  [1.03, 1.41] | **-0.23**  **[-0.41, -0.04]** |
| Participant 4 | Female | 21 | White | 0.48  [-0.49, 1.45] | 4.65  [4.02, 5.44] | 5.38  [5.11, 5.65] | 1.26  [1.09, 1.49] | 0.12  [-0.06, 0.28] |  | 1.13  [1.09, 1.16] | 0.16  [0.14, 0.19] | 5.89  [5.55, 6.24] | 1.57  [1.34, 1.86] | -0.07  [-0.23, 0.12] |
| Participant 5 | Female | 19 | White | 2.75  [1.31, 4.17] | 6.95  [6.1, 7.99] | 4.97  [4.39, 5.53] | 2.84  [2.49, 3.26] | 0.06  [-0.01, 0.16] |  | 0.96  [0.93, 0.99] | 0.13  [0.11, 0.15] | 5.41  [4.86, 5.95] | 2.61  [2.27, 3.02] | -0.11  [-0.25, 0.03] |
| Participant 6 | Female | 18 | Multiracial | -0.52  [-2.05, 1.01] | 7.38  [6.46, 8.49] | 5.61  [5.20, 6.02] | 1.85  [1.59, 2.19] | 0.06  [-0.05, 0.17] |  | 1.26  [1.20, 1.32] | 0.28  [0.24, 0.32] | 5.75  [5.33, 6.16] | 2.04  [1.77, 2.37] | -0.08  [-0.18, 0.02] |
| Participant 7 | Female | 19 | Black | -0.11  [-1.11, 0.9] | 4.50  [3.82, 5.35] | 4.61  [4.35, 4.87] | 1.09  [1.00, 1.29] | 0.07  [-0.15, 0.26] |  | 1.03  [1.00, 1.06] | 0.13  [0.11, 0.15] | 4.53  [4.19, 4.87] | 1.59  [1.36, 1.87] | -0.11  [-0.28, 0.08] |
| Participant 8 | Female | 18 | Indian | 2.15  [0.81, 3.47] | 6.23  [5.43, 7.23] | 4.45  [3.90, 5.01] | 2.62  [2.27, 3.04] | 0.07  [-0.02, 0.18] |  | 0.84  [0.81, 0.87] | 0.12  [0.10, 0.14] | 5.49  [5.03, 5.96] | 2.22  [1.95, 2.55] | **-0.37**  **[-0.52, -0.19]** |
| Participant 9 | Male | 18 | Middle-Eastern | -2.64  [-3.56, -1.69] | 3.96  [3.39, 4.71] | 6.22  [5.81, 6.63] | 1.78  [1.53, 2.1] | **0.28**  **[0.10, 0.47]** |  | 0.70  [0.68, 0.72] | 0.10  [0.08, 0.12] | 6.06  [5.66, 6.47] | 1.94  [1.68, 2.27] | -0.16  [-0.33, 0.03] |
| Participant 10 | Female | 18 | White | -0.05  [-1.31, 1.21] | 6.24  [5.46, 7.20] | 5.48  [5.11, 5.86] | 1.79  [1.55, 2.09] | 0.11  [-0.01, 0.24] |  | 0.70  [0.68, 0.73] | 0.11  [0.10, 0.13] | 5.73  [5.34, 6.12] | 1.84  [1.59, 2.16] | -0.11  [-0.28, 0.08] |
| Participant 11 | Female | 19 | White | 1.25  [0.15, 2.37] | 5.49  [4.78, 6.38] | 5.10  [4.55, 5.65] | 2.87  [2.52, 3.29] | 0.02  [-0.09, 0.11] |  | 1.30  [1.26, 1.34] | 0.17  [0.14, 0.20] | 5.28  [4.71, 5.86] | 2.78  [2.44, 3.21] | -0.08  [-0.20, 0.03] |
| Participant 12 | Female | 18 | Black | 2.03  [0.72, 3.33] | 6.25  [5.47, 7.23] | 5.68  [5.26, 6.09] | 1.92  [1.66, 2.25] | **0.11**  **[0.00, 0.24]** |  | 0.82  [0.79, 0.85] | 0.14  [0.12, 0.16] | 5.76  [5.32, 6.21] | 2.13  [1.86, 2.47] | **-0.25**  **[-0.41, -0.10]** |
| Participant 13 | Female | 19 | White | 0.34  [-0.99, 1.67] | 5.53  [4.72, 6.57] | 6.07  [5.71, 6.42] | 1.39  [1.16, 1.68] | **0.17**  **[0.01, 0.35]** |  | 1.18  [1.15, 1.21] | 0.15  [0.13, 0.17] | 6.10  [5.71, 6.50] | 1.91  [1.65, 2.23] | -0.05  [-0.2, 0.12] |
| Participant 14 | Female | 18 | White | 0.30  [-0.68, 1.29] | 4.76  [4.13, 5.54] | 5.73  [5.35, 6.1] | 1.80  [1.56, 2.10] | 0.13  [-0.01, 0.28] |  | 0.74  [0.72, 0.77] | 0.09  [0.08, 0.11] | 6.07  [5.66, 6.48] | 1.94  [1.67, 2.27] | -0.10  [-0.28, 0.10] |
| Participant 15 | Female | 18 | White | 2.46  [0.86, 4.08] | 8.51  [7.55, 9.66] | 5.58  [5.27, 5.89] | 1.46  [1.26, 1.71] | 0.07  [-0.04, 0.18] |  | 1.24  [1.19, 1.28] | 0.20  [0.17, 0.23] | 5.83  [5.50, 6.16] | 1.54  [1.32, 1.81] | -0.06  [-0.20, 0.11] |
| Participant 16 | Female | 19 | White | -0.95  [-2.19, 0.29] | 5.66  [4.89, 6.62] | 5.22  [4.74, 5.70] | 2.22  [1.92, 2.60] | 0.10  [-0.02, 0.22] |  | 1.11  [1.07, 1.16] | 0.20  [0.17, 0.23] | 5.21  [4.77, 5.65] | 2.19  [1.91, 2.53] | -0.10  [-0.22, 0.02] |
| Participant 17 | Female | 18 | White | 1.08  [0.18, 1.97] | 4.18  [3.6, 4.93] | 5.65  [5.22, 6.07] | 2.04  [1.77, 2.37] | 0.09  [-0.06, 0.24] |  | 1.25  [1.21, 1.29] | 0.18  [0.16, 0.21] | 6.06  [5.65, 6.46] | 1.93  [1.68, 2.25] | **-0.20**  **[-0.34, -0.06]** |
| Participant 18 | Female | 18 | White | -1.48  [-2.75, -0.21] | 5.71  [4.91, 6.70] | 4.63  [4.14, 5.14] | 2.23  [1.92, 2.63] | 0.04  [-0.09, 0.15] |  | 1.10  [1.07, 1.14] | 0.16  [0.14, 0.19] | 5.02  [4.55, 5.49] | 2.21  [1.93, 2.57] | **-0.20**  **[-0.35, -0.06]** |
| Participant 19 | Male | 19 | Asian | -0.99  [-1.95, -0.02] | 4.49  [3.87, 5.26] | 6.05  [5.64, 6.45] | 1.91  [1.66, 2.23] | **0.16**  **[0.01, 0.31]** |  | 0.82  [0.80, 0.84] | 0.09  [0.08, 0.11] | 6.40  [5.98, 6.82] | 1.99  [1.72, 2.33] | -0.18  [-0.35, 0.02] |
| Participant 20 | Male | 20 | Asian | -0.66  [-1.73, 0.41] | 4.45  [3.76, 5.35] | 5.02  [4.65, 5.38] | 1.49  [1.26, 1.79] | 0.10  [-0.09, 0.27] |  | 1.51  [1.44, 1.57] | 0.29  [0.26, 0.33] | 5.59  [5.23, 5.95] | 1.66  [1.43, 1.96] | -0.06  [-0.17, 0.06] |
| Participant 21 | Male | 19 | White | -1.01  [-1.76, -0.26] | 3.52  [3.03, 4.15] | 5.60  [5.15, 6.06] | 2.22  [1.94, 2.58] | 0.13  [-0.02, 0.29] |  | 0.68  [0.66, 0.70] | 0.09  [0.08, 0.11] | 5.53  [5.03, 6.03] | 2.56  [2.26, 2.94] | **-0.22**  **[-0.37, -0.06]** |
| Participant 22 | Male | 19 | White | -2.76  [-3.65, -1.84] | 4.33  [3.77, 5.03] | 4.80  [4.50, 5.10] | 1.45  [1.26, 1.69] | **0.28**  **[0.11, 0.44]** |  | 1.06  [1.02, 1.09] | 0.15  [0.13, 0.18] | 5.25  [4.75, 5.75] | 2.27  [1.97, 2.65] | **-0.23**  **[-0.4, -0.08]** |
| Participant 23 | Female | 18 | White | -0.17  [-1.23, 0.9] | 4.9 [4.22, 5.76] | 5.27  [4.79, 5.75] | 2.24  [1.94, 2.62] | 0.12  [-0.01, 0.25] |  | 1.00  [0.97, 1.04] | 0.15  [0.13, 0.17] | 5.31  [4.88, 5.74] | 1.97  [1.72, 2.29] | **-0.35**  **[-0.51, -0.17]** |
| Participant 24 | Female | 20 | White | 0.35  [-0.84, 1.55] | 5.31  [4.56, 6.25] | 5.30  [4.78, 5.82] | 2.35  [2.03, 2.76] | **0.11**  **[0.00, 0.25]** |  | 0.95  [0.92, 0.99] | 0.15  [0.13, 0.18] | 5.53  [5.04, 6.02] | 2.41  [2.11, 2.78] | -0.14  [-0.27, 0.00] |
| Participant 25 | Female | 18 | White | -1.16  [-2.07, -0.24] | 4.42  [3.82, 5.18] | 5.35  [4.79, 5.91] | 2.93  [2.58, 3.36] | 0.03  [-0.10, 0.13] |  | 0.88  [0.86, 0.91] | 0.10  [0.08, 0.11] | 5.83  [5.32, 6.34] | 2.71  [2.39, 3.09] | -0.12  [-0.27, 0.04] |
| Participant 26 | Female | 21 | White | -1.00  [-1.91, -0.08] | 4.45  [3.85, 5.21] | 5.37  [5.08, 5.65] | 1.35  [1.16, 1.59] | 0.15  [-0.02, 0.32] |  | 0.74  [0.71, 0.77] | 0.13  [0.11, 0.15] | 5.32  [4.99, 5.65] | 1.57  [1.35, 1.84] | **-0.20**  **[-0.37, -0.02]** |
| Participant 27 | Female | 19 | Indian | 2.31  [1.06, 3.56] | 6.25  [5.50, 7.18] | 5.38  [5.04, 5.72] | 1.60  [1.38, 1.87] | **0.17**  **[0.04, 0.32]** |  | 0.93  [0.90, 0.96] | 0.14  [0.12, 0.17] | 5.94  [5.68, 6.21] | 1.22  [1.06, 1.45] | **-0.27**  **[-0.44, -0.07]** |
| Participant 28 | Female | 20 | White | 1.47  [0.08, 2.84] | 6.75  [5.92, 7.78] | 5.18  [4.94, 5.41] | 1.04  [1.00, 1.16] | 0.16  [-0.01, 0.33] |  | 1.33  [1.27, 1.38] | 0.23  [0.20, 0.27] | 5.18  [4.90, 5.46] | 1.25  [1.07, 1.48] | **-0.19**  **[-0.34, -0.03]** |
| Participant 29 | Male | 19 | White | 1.41  [0.24, 2.59] | 5.83  [5.10, 6.72] | 5.17  [4.89, 5.44] | 1.29  [1.12, 1.52] | **0.16**  **[0.01, 0.32]** |  | 0.94  [0.91, 0.98] | 0.15  [0.13, 0.18] | 5.48  [5.14, 5.83] | 1.58  [1.35, 1.88] | -0.14  [-0.30, 0.04] |
| Participant 30 | Female | 19 | White | 1.85  [0.83, 2.86] | 4.85  [4.20, 5.67] | 5.79  [5.45, 6.12] | 1.61  [1.39, 1.89] | 0.03  [-0.15, 0.17] |  | 0.77  [0.75, 0.79] | 0.10  [0.08, 0.11] | 5.32  [4.94, 5.71] | 1.83  [1.59, 2.14] | **-0.29**  **[-0.46, -0.11]** |
| Participant 31 | Male | 20 | White | 1.12  [0.09, 2.14] | 4.82  [4.16, 5.64] | 6.52  [6.08, 6.96] | 2.11  [1.83, 2.46] | 0.02  [-0.14, 0.15] |  | 1.00  [0.96, 1.04] | 0.18  [0.16, 0.21] | 6.13  [5.69, 6.57] | 2.26  [1.98, 2.6] | **-0.13**  **[-0.26, -0.01]** |
| Participant 32 | Female | 18 | Asian | 0.43  [-0.71, 1.55] | 5.39  [4.69, 6.26] | 6.00  [5.62, 6.39] | 1.81  [1.56, 2.12] | **0.16**  **[0.03, 0.32]** |  | 1.18  [1.13, 1.24] | 0.20  [0.17, 0.24] | 6.81  [6.35, 7.26] | 1.77  [1.49, 2.15] | -0.11  [-0.26, 0.04] |
| Participant 33 | Female | 18 | Asian | -1.05  [-2.08, -0.02] | 4.90  [4.24, 5.72] | 5.81  [5.52, 6.09] | 1.29  [1.11, 1.52] | **0.19**  **[0.02, 0.35]** |  | 0.94  [0.91, 0.98] | 0.15  [0.13, 0.18] | 5.61  [5.27, 5.94] | 1.57  [1.34, 1.85] | **-0.20**  **[-0.37, -0.03]** |
| Participant 34 | Female | 19 | White | 1.12  [0.23, 2.01] | 4.26  [3.67, 5.00] | 5.29  [4.94, 5.64] | 1.70  [1.47, 1.99] | 0.02  [-0.17, 0.17] |  | 0.75  [0.73, 0.78] | 0.11  [0.09, 0.13] | 5.60  [5.20, 5.99] | 1.93  [1.67, 2.25] | -0.07  [-0.24, 0.12] |
| Participant 35 | Female | 18 | White | -0.44  [-1.51, 0.63] | 5.24  [4.56, 6.09] | 5.47  [5.08, 5.85] | 1.87  [1.62, 2.19] | 0.08  [-0.06, 0.21] |  | 1.05  [1.02, 1.09] | 0.14  [0.12, 0.17] | 5.97  [5.54, 6.40] | 2.05  [1.77, 2.39] | -0.11  [-0.26, 0.05] |
| Participant 36 | Female | 18 | White | -1.22  [-2.13, -0.29] | 4.19  [3.58, 4.97] | 5.51  [5.27, 5.74] | 1.05  [1.00, 1.21] | 0.04  [-0.19, 0.25] |  | 0.93  [0.90, 0.95] | 0.11  [0.09, 0.13] | 5.23  [4.91, 5.55] | 1.41  [1.20, 1.69] | -0.11  [-0.31, 0.11] |
| Participant 37 | Male | 21 | White | 3.59  [2.32, 4.83] | 5.62  [4.85, 6.58] | 3.17  [2.70, 3.67] | 2.03  [1.73, 2.4] | 0.09  [-0.03, 0.22] |  | 1.06  [1.02, 1.1] | 0.18  [0.15, 0.20] | 4.33  [3.97, 4.69] | 1.73  [1.51, 2.00] | **-0.28**  **[-0.44, -0.13]** |
| Participant 38 | Male | 19 | Hispanic | 1.17  [-0.12, 2.45] | 6.46  [5.66, 7.43] | 5.74  [5.48, 6.00] | 1.23  [1.06, 1.45] | 0.05  [-0.11, 0.19] |  | 0.90  [0.86, 0.93] | 0.16  [0.14, 0.18] | 5.75  [5.44, 6.07] | 1.51  [1.32, 1.75] | **-0.37**  **[-0.52, -0.20]** |
| Participant 39 | Female | 19 | White | 1.10  [0.03, 2.18] | 5.33  [4.65, 6.17] | 5.60  [5.23, 5.96] | 1.74  [1.51, 2.03] | **0.14**  **[0.01, 0.29]** |  | 1.48  [1.44, 1.52] | 0.16  [0.14, 0.19] | 5.87  [5.42, 6.32] | 2.18  [1.90, 2.53] | -0.12  [-0.26, 0.01] |
| Participant 40 | Female | 20 | White | -0.53  [-1.67, 0.61] | 5.44  [4.72, 6.33] | 5.27  [4.94, 5.61] | 1.54  [1.33, 1.82] | 0.09  [-0.06, 0.23] |  | 1.19  [1.15, 1.23] | 0.18  [0.16, 0.21] | 5.32  [4.98, 5.66] | 1.64  [1.41, 1.91] | **-0.18**  **[-0.32, -0.03]** |
| Participant 41 | Male | 21 | Asian | 0.97  [0.15, 1.78] | 3.88  [3.33, 4.58] | 5.38  [5.08, 5.69] | 1.44  [1.24, 1.70] | 0.07  [-0.13, 0.24] |  | 0.67  [0.65, 0.69] | 0.09  [0.08, 0.11] | 5.36  [4.99, 5.73] | 1.78  [1.53, 2.09] | -0.17  [-0.35, 0.03] |
| Participant 42 | Male | 21 | Multiracial | 1.95  [0.91, 2.98] | 4.78  [4.12, 5.61] | 6.12  [5.58, 6.66] | 2.62  [2.28, 3.03] | 0.06  [-0.06, 0.17] |  | 0.68  [0.66, 0.70] | 0.09  [0.07, 0.10] | 6.10  [5.55, 6.64] | 2.69  [2.36, 3.10] | -0.16  [-0.32, 0.02] |
| Participant 43 | Female | NA | NA | -0.45  [-1.84, 0.95] | 6.81  [5.97, 7.83] | 6.18  [5.75, 6.60] | 1.96  [1.70, 2.3] | **0.13**  **[0.02, 0.27]** |  | 1.20  [1.15, 1.24] | 0.19  [0.17, 0.22] | 6.75  [6.35, 7.13] | 1.76  [1.51, 2.07] | **-0.16**  **[-0.3, -0.01]** |
| Participant 44 | Male | 20 | White | 0.08  [-1.09, 1.25] | 5.80  [5.06, 6.7] | 5.22  [4.81, 5.62] | 1.96  [1.70, 2.28] | **0.12**  **[0.01, 0.26]** |  | 1.36  [1.30, 1.41] | 0.24  [0.21, 0.28] | 5.64  [5.19, 6.09] | 2.17  [1.89, 2.53] | -0.09  [-0.20, 0.01] |
| Participant 45 | Male | 19 | Asian | -0.43  [-1.37, 0.53] | 4.19  [3.57, 4.99] | 6.31  [5.99, 6.63] | 1.39  [1.18, 1.66] | 0.11  [-0.08, 0.28] |  | 0.66  [0.63, 0.69] | 0.10  [0.08, 0.12] | 6.14  [5.68, 6.61] | 1.89  [1.60, 2.26] | **-0.24**  **[-0.43, -0.04]** |
